# Using sea-ice to calibrate a dynamic trophic model for the Western Antarctic Peninsula

**DOI:** 10.1371/journal.pone.0214814

**Published:** 2019-04-02

**Authors:** Adrian Dahood, George M. Watters, Kim de Mutsert

**Affiliations:** 1 Department of Environmental Science and Policy, George Mason University, Fairfax, Virginia, United States of America; 2 Antarctic Ecosystem Research Division, Southwest Fisheries Science Center, National Marine Fisheries Service, National Oceanic and Atmospheric Administration, La Jolla, California, United States of America; 3 Institute of Marine Sciences, University of California Santa Cruz, Santa Cruz, California, United States of America; Victoria University of Wellington, NEW ZEALAND

## Abstract

The pelagic ecosystems of the Western Antarctic Peninsula are dynamic and changing rapidly in the face of sustained warming. There is already evidence that warming may be impacting the food web. Antarctic krill, *Euphausia superba*, is an ice-associated species that is both an important prey item and the target of the only commercial fishery operating in the region. The goal of this study is to develop a dynamic trophic model for the region that includes the impact of the sea-ice regime on krill and krill predators. Such a model may be helpful to fisheries managers as they develop new management strategies in the face of continued sea-ice loss. A mass balanced food-web model (Ecopath) and time dynamic simulations (Ecosim) were created. The Ecopath model includes eight currently monitored species as single species to facilitate its future development into a model that could be used for marine protected area planning in the region. The Ecosim model is calibrated for the years 1996–2012. The successful calibration represents an improvement over existing Ecopath models for the region. Simulations indicate that the role of sea ice is both central and complex. The simulations are only able to recreate observed biomass trends for the monitored species when metrics describing the sea-ice regime are used to force key predator-prey interactions, and to drive the biomasses of Antarctic krill and the fish species *Gobionotothen gibberifrons*. This model is ready to be used for exploring results from sea-ice scenarios or to be developed into a spatial model that informs discussions regarding the design of marine protected areas in the region.

## Introduction

The Western Antarctic Peninsula (WAP) is one of the most rapidly warming regions on Earth [1–3] with an average air temperature increase at the surface of approximately 5-6° C since 1960 [4, 5]. Long-term datasets describing the sea-ice regime illustrate significant changes in response to this prolonged warming and increasing number of days where the air temperature exceeds freezing [4]. “Permanent” ice shelves that rest over the sea have been retreating for the past 20 years, and winter sea-ice concentration and extent are both decreasing [1, 2]. Sea ice is forming later in the season and retreating earlier [1, 4]. Throughout the Antarctic Peninsula region the winter-ice season has decreased by one to two days per year on average [4], and the total the sea-ice season shrank by 92 days from 1979–80 to 2012–13 [2]. Sea ice is critically important in structuring WAP marine ecosystems [1, 2].

Marine food webs in the WAP are often described as krill-centric [6–8]. Diet studies of numerous predators in the region indicate that Antarctic krill (*Euphausia superba*, hereafter krill) is an important prey species for a wide variety of predators [9]. Krill are the target of the largest (by tonnage) Antarctic fishery, with about 155,000 t yr^-1^ landed from around the WAP, within Statistical Subarea 48.1 as defined by the Commission for the Conservation of Antarctic Marine Living Resources (CCAMLR) [10, 11]. Krill are patchily distributed, and krill abundance can vary by orders of magnitude [12–15]. These realities imply that the role of krill in the food web can vary both spatially and temporally.

There is evidence that warming around the WAP has altered the food web [1]. In areas that have experienced sustained warming and associated ice loss, salps (*Salpa thompsoni*) may replace krill as the dominant phytoplankton consumer [16]. Top predators such as Adélie (*Pygoscelis adeliae*) and chinstrap (*P*. *antarcticus*) penguins may be declining throughout the WAP, and these declines are correlated with increasing temperatures and ice loss [17]. Abundant piscine predators like *Gobionotothen gibberifrons* also appear to have experienced significant declines yet it is unclear what is causing these trends [18]. The changing sea-ice regime is likely influencing predator-prey dynamics and population dynamics at all levels of the food web. Marine-resource managers for the region may find it useful to explore how changes in the sea-ice regime and consequent effects on the food web could impact harvested and monitored species before making changes to fisheries-management strategies. Such explorations may be particularly helpful in light of ongoing discussions regarding the design of marine protected areas (MPAs) in the region (paragraphs 5.63–5.69 of [19]).

The software package Ecopath with Ecosim (EwE) was designed to facilitate the creation of dynamic food-web models that can be used to aid the development of fisheries-management strategies, including the development of MPAs [20, 21]. To explore such options, EwE implements a mass balanced food-web model (Ecopath) and time dynamic simulations (Ecosim) that aim to recreate observed biomass trends for key components in the ecosystem. There are three published EwE models that overlap in whole or in part with the WAP: Cornejo-Donoso and Antezana [22], Ballerini et al. [7] and Suprenand and Ainsworth [23]. Two other EwE models have been made available in the grey literature [24, 25]. All five sets of authors produced mass balanced Ecopath models, but none of the models were calibrated by fitting to time-series observations in Ecosim. The previous models do not recreate observed trends in the biomasses of several monitored species. Furthermore, because species are aggregated, the existing models do not facilitate consideration of species-specific responses to environmental change and alternative management decisions. These existing models are poorly suited to be used as decision support tools for the MPA process.

The objectives of this study are to1) develop a mass balanced food-web model that explicitly describes the dynamics of monitored and declining species and 2) calibrate the model by fitting time dynamic simulations to observed trends in the biomasses of species that are monitored in the WAP. Having accomplished these objectives, we assert the model would be suitable for further development into a spatial model to be used as a decision support tool within the CCAMLR process to designate MPAs. The work presented here evaluates the extent to which changes in sea-ice cover explain observed variations in species biomass by including the sea-ice regime as an environmental driver. We note that the relationship between krill and sea ice is uncertain [14] and that other environmental drivers may influence krill and its predators. The work presented here focuses on the role of sea ice in structuring the food web in the WAP as one plausible hypothesis to explain temporal patterns of biomass for eight monitored species.

## Methods

### Study area

**Fig 1:**
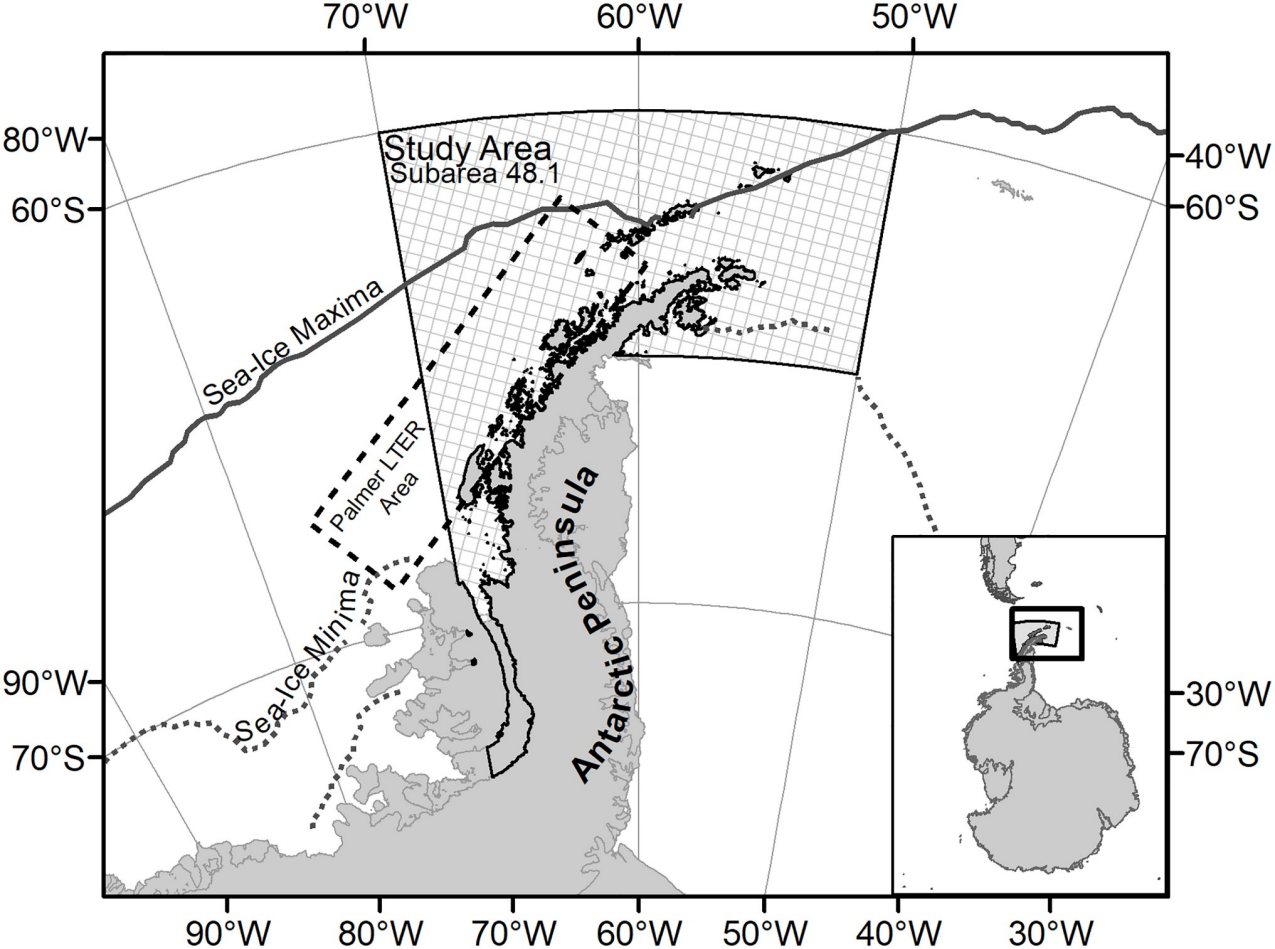
Study area detail. Map, including reticules, was created using ArcMap 10.6. The Antarctic continent shapefile is freely available from the Antarctic Digital Database [26], the boundary of Statistical Area 48.1 is freely available from CCAMLR’s online GIS [27]. The Natural Earth (https://www.naturalearthdata.com/) provides public domain shapefiles of the countries of the world. The polygon bounding the Palmer LTER Study Area was drawn to bound the stations identified in the Palmer LTER Basic Grid[28] The displayed sea-ice maxima is a climatology (1981–2010) describing the median location of the sea edge in the month of August as made available from the National Snow and Ice Data Center [29]. The displayed sea-ice minima is a climatology (1981–2010) describing the median location of the sea ice edge in February as made available from the National Snow and Ice Data Center [29].

The study area was Statistical Subarea 48.1 as defined by the Commission for the Conservation of Antarctic Marine Living Resources (CCAMLR). This is a region of the southwest Atlantic that includes the WAP and South Shetland Islands [10]. Subarea 48.1 has an area of approximately 630,279 km^2^ [9] and is south of South America (Fig 1). The only commercial fishery currently operating in Statistical Subarea 48.1 is the krill fishery, which removed an average of approximately 51,000 tonnes of krill per year in the 1990s and 33,000 tonnes of krill per year in the early 2000s [10]. The krill catch in Statistical Subarea 48.1 has been increasing with catch exceeding 150,000 tonnes in 5 of the last eight years [10].

#### Mass-balanced food-web model (Ecopath)

Ecopath with Ecosim (EwE) is open source, freely available software that has been used to model ecosystems worldwide [20, 21]. Ecopath creates a mass balanced model of the food web assuming that predation, fishing pressure, and competition are critical to structuring the community [20, 21].

Ecopath relies on two master equations to parameterize the model. As described in Christensen and Walters (2004), the first equation describes the production term, *P*_*i*_.

Pi=Yi+M2i×Bi+Ei+BAi+M0i×Bi (1)

For each species i, *Y*_*i*_ is the total fishery catch, *M*2_*i*_ is the instantaneous predation rate, *B*_*i*_ is the biomass, *E*_*i*_ is the net migration rate, *BA*_*i*_ is biomass accumulation, and *M*0_*i*_ is ‘other’ mortality.

The second master equation describes the energy balance of each group such that

Consumption=production+respiration+unassimilatedfood (2)

This equation requires that the consumption of any one model group is always less than or equal to its production, thus ensuring energy balance within each group [20].

For this study, the food web of the WAP was simplified into 35 single- and multi-species groups, and these groups were selected to represent all levels of the food web, from detritus to apex predators. (see S1 File for group definitions). Due to the importance of krill in the region [2, 7, 8], special attention was paid to krill and monitored krill predators. Species that the CCAMLR have designated as indicator species [30, 31] were represented as single-species groups. It was our intention that this model eventually be developed into a spatial model (Ecospace) that can be used to inform the development of marine protected areas in the region. All currently monitored species seem likely to feature in a research and monitoring plan associated with a new MPA and were modeled as single species. Other species, such as non-krill zooplankton, phytoplankton, and fishes for which relatively little data exist, were combined into multi-species functional groups.

#### Specification of Ecopath biomass

The base year of the model was 1996. Initial biomass estimates for all consumer groups reflected data collected during the period 1992–2002, and for many groups only a single estimate was available during this time frame (S2 File). For large krill, multiple density estimates are available during this time period and we averaged densities for the years 1996–2001 to determine the biomass to input to Ecopath. Data on the biomass of primary producers in the region are scarce and region-wide estimates are not available. While satellite imagery describing chlorophyll concentrations is available nine months of the year [32], measurements of chlorophyll concentrations do not capture ice algae and may not correlate well with *in situ* measurements of phytoplankton biomass [33]. Rather than introduce additional complexity and uncertainty by attempting to estimate the biomasses of the phytoplankton and ice-algae functional groups from remotely sensed imagery, we used the model itself to calculate biomass for primary producers. This was accomplished by setting the ecotrophic efficiency (EE), the proportion of production of any given model group utilized in the system (see equation 6 of [20]), and allowing the model to calculate the biomass required to balance this proportion of utilization. This was the same approach used by Ballerini et al. [7]. Following the advice of Heymans et al. [34], the EE for primary producers in this largely pelagic ecosystem was set to 0.5 [33]. For all other groups, we input biomasses and used the model to calculate the EEs. Fisheries catch data are regularly reported to the CCAMLR [10], and the average krill catch for the years 1995–2001, which represented catches near the base year of the model, was included as “landings” in Ecopath. We did not include discards as discards are not clearly identified in the publicly available CCAMLR data [10].

The abundances of two species of whales are known to be increasing in the study area [35, 36]. However, while estimates of the rate of increase have been presented, no reliable times series of abundance are available. Humpback whales (*Megaptera novaeangliae*) have experienced an estimated average population growth for the region of 4.5% per year with a 95% confidence interval of -2.9% to 12.3% [35]. Initially, we used a biomass accumulation term of 4.5%, but the model failed to balance if the biomass accumulation term for humpback whales was greater than 3.9%, so we used this later value. While annual population-growth rates have not been published for fin whales (*Balaenoptera physalus*) in the study region, reported sightings data [36] reveal that fin whale sightings have increased at a similar, but slightly lesser rate. A biomass accumulation term of 2.9% per year was used for Fin whales.

#### Specification of Ecopath production to biomass ratio

The production to biomass ratio (P/B) describes the turnover rate, or rate at which a trophic group can replace itself. This rate is poorly described for many species at lower trophic levels. Due to lack of data, the P/B ratios for primary producers, micro-, meso-, and macro- zooplankton, salps, and benthic invertebrates were adopted from previously published models. Ballerini et al. [7] estimated phytoplankton productivity from satellite imagery; the estimated P/B for the “other euphausiid” functional group was from studies conducted in Japan [37]; and the estimated P/B value for salps was from Pakhomov [38].

Krill were modeled as a multi-stanza group, with one stanza for animals younger than 24 months (small krill) and a second for animals older than 24 months (large krill). Instead of P/B, base mortality (Z) was input for each stanza [20]. The multi-stanza approach assumes that body growth for the species follows a von Bertalanffy curve and that the species’ population, as a whole, has reached a stable age-size distribution [20]. These assumptions seem valid for the Antarctic krill population [39, 40]. A recent review of published mortality rates for krill indicates that temperature, age composition of the population, and sub region where the krill are sampled can significantly influence estimated mortality rates; estimates range from 0.38 to 1.22 [41]. Models currently used to inform the management of the krill fishery use a natural mortality value of 0.8 [42]. Krill catches, approximately 210,000 tonnes for the entire fishery during the period 1992–2002 [10] are low compared to consumption by predators [43], so the natural mortality rate is a good approximation of Z for krill. We used the mortality value used to inform management of the krill fishery, 0.8, for both large and small krill. This value, and the von Bertalanffy curvature constant (K) of 0.440 are derived from the work of Rosenberg et al. [40] and of Candy and Kawaguchi [39].

Hill et al. [9] provide a compilation of all estimates of P/B ratios for fishes. The P/B value for myctophids ranges between 0.86–1.14. The value of 1.1 was used in the current model. Similarly, Hill et al. [9] note that the P/Bs of fishes living on the continental shelf range from 0.19–0.60 and recommend a value of 0.46, which was adopted here. A natural mortality rate of 0.29 is recognized as the best estimate for *Notothenia rossii* [18] and was used in our model. Iverson [44] estimated that the pre-exploitation natural mortality rate of *Champsocephalus gunnari* ranged between 0.23–0.96, and models used to inform fisheries management for this species use the midrange value of 0.48 [9]. We also used the value of 0.48. A specific P/B value for *G*. *gibberifrons* could not be found in the literature. The species is included in the Hill et al. [9] assessment of the mortality for shelf-associated fishes, and, therefore, our model used a P/B of 0.46 for *G*. *gibberifrons*.

For upper level predators, the P/B ratio can be represented by the annual rate of adult natural mortality [7, 45], and this is a commonly published parameter (S3 File). For all marine mammal and penguin groups, our model used published values of survival or mortality. Following Ballerini et al. [7], the P/B value for flying birds was calculated using a weighted average of annual survival for each species included in the functional group. Weights were proportional to the relative abundances of the species as described by Ribic et al. [46].

#### Specification of Ecopath production to consumption ratio

To create a mass balanced model, Ecopath uses an estimate of consumption (Eqs 1 and 2). This estimate is often input to the model as either a production to consumption (P/Q) ratio or a consumption to biomass (Q/B) ratio. The P/Q ratio can be calculated as growth efficiency, or the product of the assimilation efficiency (AE) and production efficiency [PE; 7]. Published AE values exist for many groups (S4 File). The PE values were derived from Townsend et al. [47]. Consistent with Ballerini et al. [7] we calculated Q/B ratios by dividing group specific estimates of P/Q into group specific estimate of P/B.

#### Specification of Ecopath diet matrix

The diet matrix describes trophic interactions among all species and functional groups in the model. Cannibalism was not allowed to occur for any group as it can cause instability [7, 48]. We note that for the multispecies functional groups where cannibalism is most likely to occur, there are not sufficient data to support further subdivision of the groups or creation of multi stanzas for the group to circumvent within group cannibalism. The diet matrix was informed by published diet-composition studies and publicly available reports of prey choices. Except for sperm whales, diet data were sourced from studies conducted in the Antarctic. Diet studies referenced include gut content analyses, visual observation of prey consumption, and stable isotope analysis. Sources for the diet matrix and notes describing how the diets were adapted from the values provided in the published literature are provided in S5 File.

#### Balancing the Ecopath model

We collated the data described above to create a mass balanced food-web model for the WAP, specifically Statistical Subarea 48.1. When the model was initially implemented, with parameters taken directly out of the literature, EE values for several groups, including important prey species such as krill and on-shelf fish, were significantly greater than 1. Predation pressure was too high on those groups. Many of the diet studies referenced within the diet matrix have small, spatially constrained sample sizes relative to their respective populations (e.g.[49, 50, 51]). It was assumed that the diets presented in studies covering small or restricted areas accurately represent the diversity of important prey items for each species, but that the percentage of mass in the diet was uncertain. Thus, we balanced the model by adjusting the diet matrix. Starting with the diets of predators that ate the prey items with the highest EE, diets were adjusted incrementally until the model balanced. This was an iterative process during which we made small changes to diets, less than 5% at a time, and then checked the EE values for all prey items to ensure the latter parameters were less than one. Balance was achieved, and diet iterations complete, when the EEs for all groups were less than one.

### Time dynamic simulation (Ecosim)

#### Equations

Ecosim allows for time dynamic simulations of the balanced model created in Ecopath. Ecosim employs coupled differential equations that are derived from the Ecopath Master Equation [20] and are expressed as:

$$\frac{dB_{i}}{dt}=g_{i}\sum_{j}Q_{ji}-\sum_{j}Q_{ij}+I_{i}-({MO}_{i}+F_{i}+e_{i})\times B_{i}$$ (3)

Where $\frac{dB_{i}}{dt}$ is the growth rate during time *t* of the biomass of group *i*; *g*_*i*_ is the net growth efficiency; *Q*_*ji*_ represents the total consumption by predator group *i* of prey from group *j* (*Q*_*ji*_ is similar); *I*_*i*_ is the biomass immigration rate and is assumed constant over time; *MO*_*i*_ is the mortality rate that is not associated with predation; *F*_*i*_ is the fishing mortality rate; and *e*_*i*_ is the emigration rate. The net migration term is *e*_*i*_ x *B*_*i*_*—I*_*i*_ and is composed of terms that are held constant over the simulation (*I*_*i*_) and those that vary over the course of the simulation (*e*_*i*_ x *B*_*i*_) [20].

Consumption rates in Ecosim are based on a simple Lotka-Volterra predator-prey model that has been modified to include “foraging arena” characteristics [20]. The foraging-arena concept recognizes that prey can occur in states that are vulnerable to predation and states that are not. Prey shift between these states as they seek to access resources like shelters that make them safer or seek food in areas that leave them more exposed. The different vulnerabilities of the prey can affect the consumption rate by predators. [20]. Consumption rates, Q_ij_, are calculated as follows:

$$Q_{ij}=\frac{a_{ij}\times v_{ij}\times B_{i}\times B_{j}\times T_{i}\times T_{j}\times S_{ij}\times (M_{ij}/D_{j})}{v_{ij}+v_{ij}\times T_{i}\times M_{ij}+a_{ij}\times M_{ij}\times B_{j}\times S_{ij}\times (T_{j}/D_{j})}$$ (4)

Where *a*_*ij*_ is the effective search rate for prey *i* by predator *j*; *v*_*ij*_ is the vulnerability of the prey *i* to predator *j*; *B*_*i*_ is the biomass of group *i*; *T*_*i*_ is the relative feeding time of group *i*; *S*_*ij*_ is a forcing function; *M*_*ij*_ represents mediation (which is not used in this model); and *D*_*j*_ describes how handling time limits consumption rates [20]. Further information describing how forcing functions can be used to drive consumption dynamics is provided by Christensen et al. (see Equations 4 and 5 and Figure 1 in [52]).

#### Ecosim time series

Three types of data were used in the time dynamic simulations for the WAP. The first were time series that describe trends in the biomasses of eight monitored species for the years 1996–2012 and which were used to asses model fit. None of the biomass time-series data were used to force corresponding biomasses the model. Reliable, yearly time-series datasets dating back to at least the 1990s are available for five species included in the model: Antarctic fur seals, Adélie penguins, chinstrap penguins, gentoo penguins, and Antarctic krill. Less regular time-series data are available for three fishes: *N*. *rossii*, *C*. *gunnari*, and *G*. *gibberifrons* (S6 File provides sources and notes describing the biomass time-series data). The time series-data used for fur seals were collected at the colony that is responsible for approximately 80% of the pup production in the region[53]. The time-series data for the three penguin species reflect region-wide trends [17, 54]. Krill recruitment patterns are similar in terms of timing and magnitude in both the northern and southern half of the study area [41] and therefore using data collected in the northern half of the study area can be considered to reflect region wide trends. Datasets for air-breathing vertebrates represent counts of animals in discrete locations; krill data represent estimated densities from acoustic surveys (fisheries independent) and fisheries catch (fisheries dependent); and fish data are biomass estimates derived from trawl surveys. Fisheries independent measurements of krill density were used to asses fit of the model. All time-series data describing trends in biomass were included in the model as “relative biomass”, which allows the model to fit to trends for these species, without information on scale. To keep the start of the time dynamic simulations consistent with the model year, data prior to 1996 were not included in the Ecosim runs.

Times series for catches taken by the krill fishery and krill-fishing effort [10] were initially included in the model. It is possible to asses model fit for krill biomass using the catch data. However commercial fishing activity has recently concentrated in relatively confined areas defining preferred fishing grounds [11]. These data may not be representative of the region and may show patterns more related to the economics of fishing than to krill biology. Therefore, the model was fitted to the fisheries independent data. Fishing effort data, as reported to CCAMLR [10] were included in the model to capture the temporal dynamics of the fishery from 1996–2012.

The third type of data used in Ecosim simulations were time series describing environmental conditions that may have influenced biomass trends, called forcing functions. Four forcing functions were used: sea-ice area, open water area, chlorophyll *a* concentration, and observed predation mortality rate of fur seal pups. Forcing functions were applied as multipliers to impact specific predator-prey interactions, or for primary producers as a multiplier on production rate. For consumers, the multiplier can be applied to search rate, vulnerability, foraging arena area or a combination of vulnerability and arena area [20]. Forcing functions were also applied to more directly drive the biomasses of krill and *G*. *gibberifrons* using response curves to tie the increases in biomass to changing environmental conditions. The processes for evaluating applications of forcing functions and response curves are discussed in the Model Calibration section below. Due to the hypothesized importance of the sea-ice regime in influencing krill abundance and ecosystem dynamics [1, 2, 16], special attention was paid to sea-ice forcing.

**Fig 2:**
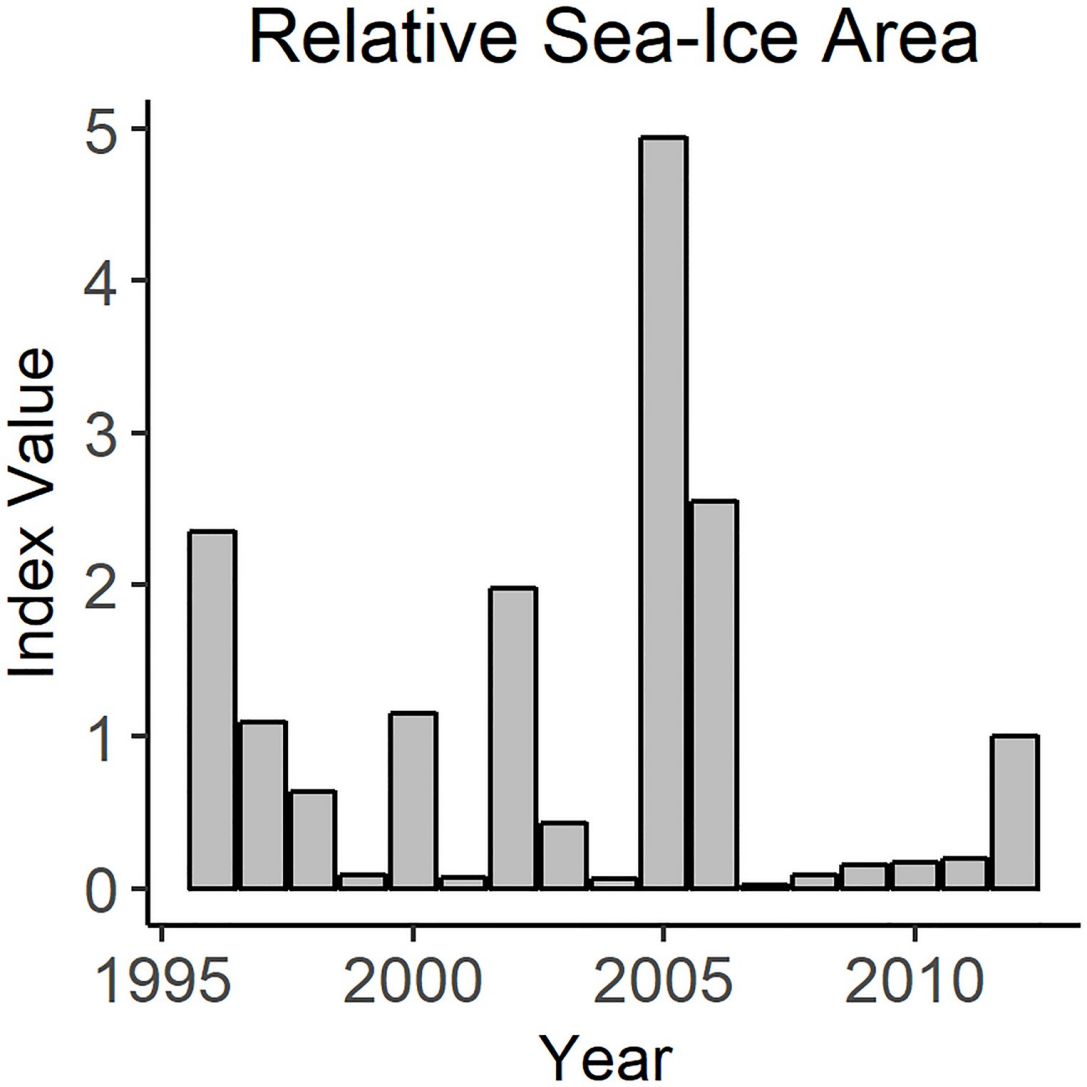
Annual sea-ice index values.

The Palmer Long Term Ecological Research program (LTER) program serves time series of monthly average sea-ice and open water areas in km^2^ for their study area (https://oceaninformatics.ucsd.edu/datazoo/catalogs/pallter/datasets/34). The sea-ice area is sensed by microwave satellite. Areas are considered “iced” when they have more than 15% ice cover [55] and a time series was created of the monthly average total iced area (in km^2^). Initially, the satellite derived, unaltered time series of total sea-ice covered area (in km^2^) for the Palmer LTER study area was incorporated in the model. However, that appeared to have little or no impact on model patterns of biomass (hereafter model results); model results did not fit the time-series data describing changes in biomass. A sea-ice index was made to identify “good ice years” and smooth some of this variability. Since winter sea ice is thought to affect Adélie penguin survivorship [56, 57] attempts were made to focus on winter ice conditions. However, the Palmer LTER sea-ice dataset does not exhibit sufficient variability in the maximum winter sea-ice area. For many years, the entire LTER study area was completely covered in ice, and thus this measure does not adequately distinguish between years. Instead, the annual summer sea-ice area minimum was used to construct the sea-ice index. The assumption that years with greater sea-ice in the summer also have greater annual ice coverage underlies our index. This may be a reasonable assumption as warmer summers are known to contribute to accelerating sea-ice loss through a positive feedback loop [2, 58, 59], and therefore cooler icier summers would not cause ice to be lost as rapidly as warmer, less icy, summers. We used annual sea-ice minima, as documented in the Palmer LTER data, that were scaled by the average value to construct the index. Years where the minimum sea-ice area was greater than average had index values greater than one; those below average had index values less than one. The scaled dataset (Fig 2) was used as the sea-ice forcing function in the model. The annual value was repeated for each monthly time step of that year, and, during the calibration process, the forcing function was applied as a multiplier to impact specific predator-prey interactions.

**Fig 3:**
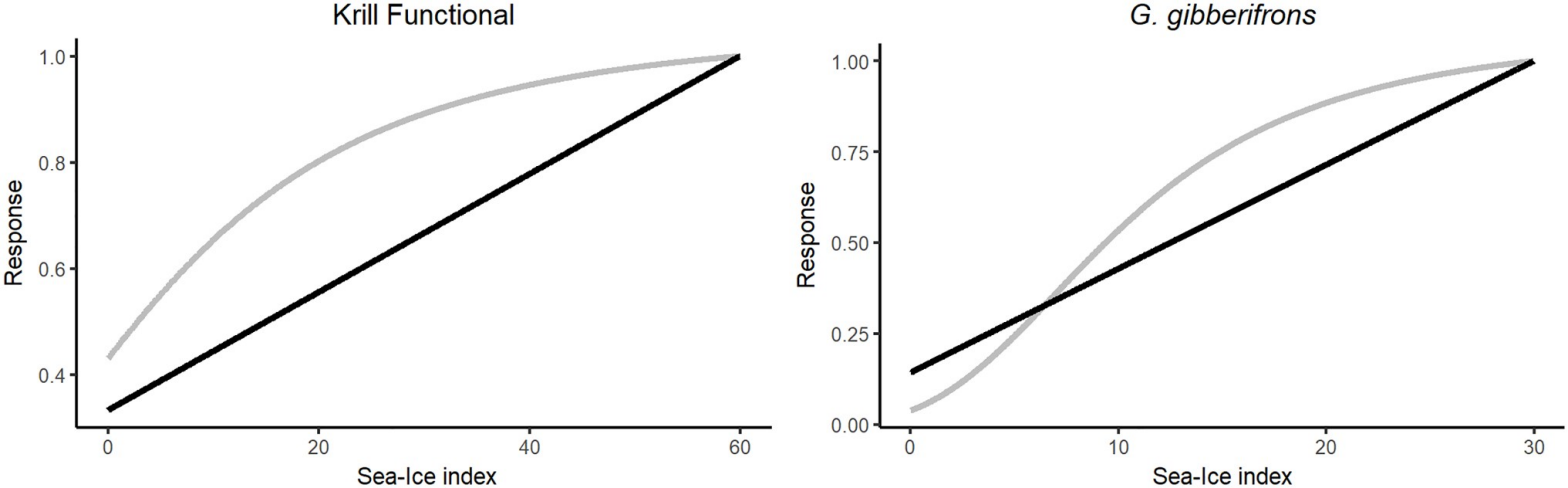
Evaluated functional responses curves. Black lines are the linear curves, grey lines represent the sigmoidal (krill) and normal (*G*. *gibberifrons*) curves.

Functional response curves were used to describe how the biomasses of krill and *G*. *gibberifrons* respond to changes in the sea-ice index. Curves were fitted for both species independently, after forcing alone failed to help the model fit the time-series data for these two species. While our understanding regarding how krill responds to sea ice conditions is still evolving [14], previous studies have shown that krill exhibit declining abundance and may be replaced by salps in areas that have experienced significant ice loss [16]. Krill recruitment is often higher following winters with greater sea-ice extent [41, 60, 61]. Data provided in the literature were not sufficient to directly construct a response curve for large krill. To further explore the hypothesis that krill generally respond positively to increased sea-ice, two curves were evaluated (Fig 3), both of which caused krill biomass to increase with the sea-ice index: linear (Ecosim parameters: start = 0; end = 60) and sigmoidal (Ecosim curve parameters: Y_zero_ = 0; Y_base_ = 1.5; Y_end_ = 5; Steep = 3).

*Gobionotothen gibberifrons* is a benthic fish that breeds in the winter, and releases pelagic eggs [62]. Available time-series data indicate a large decline in the biomass of this species. This decline was first noted in 2001, but its cause is currently unknown [18, 63]. This fish species dwells in the northern part of the study area and is not considered ice dependent [62]. However, this animal breeds in the winter and could possibly respond positively to sea ice, or oceanic conditions associated with sea ice. After applying forcing using the sea-ice index and the open water forcing function to predator-prey interactions both directly involving this species and more broadly to fit the model for the seven other species for which time series the model failed to recreate the decline as described in the biomass of *G*. *gibberifrons*. Instead the model predicted an increase in this species. In an attempt to help the model recreate the observed decline in this species, simulations were run with sea ice driving the biomass for *G*. *gibberifrons*. We note that the sea-ice index could be serving as a proxy for other unmodeled environmental conditions to which *G*. *gibberifrons* responds positively. However, using the sea-ice index as a driver of *G*. *gibberifrons* biomass resulted in the model recreating the documented decline. The simulations used curves drawn directly in Ecosim (Fig 3): linear curve (start = 0, end = 304) and normal curve (Ecosim curve parameters: SD left = 12; data width = 560; SD right = 100; mean = 24; max = 1).

**Fig 4:**
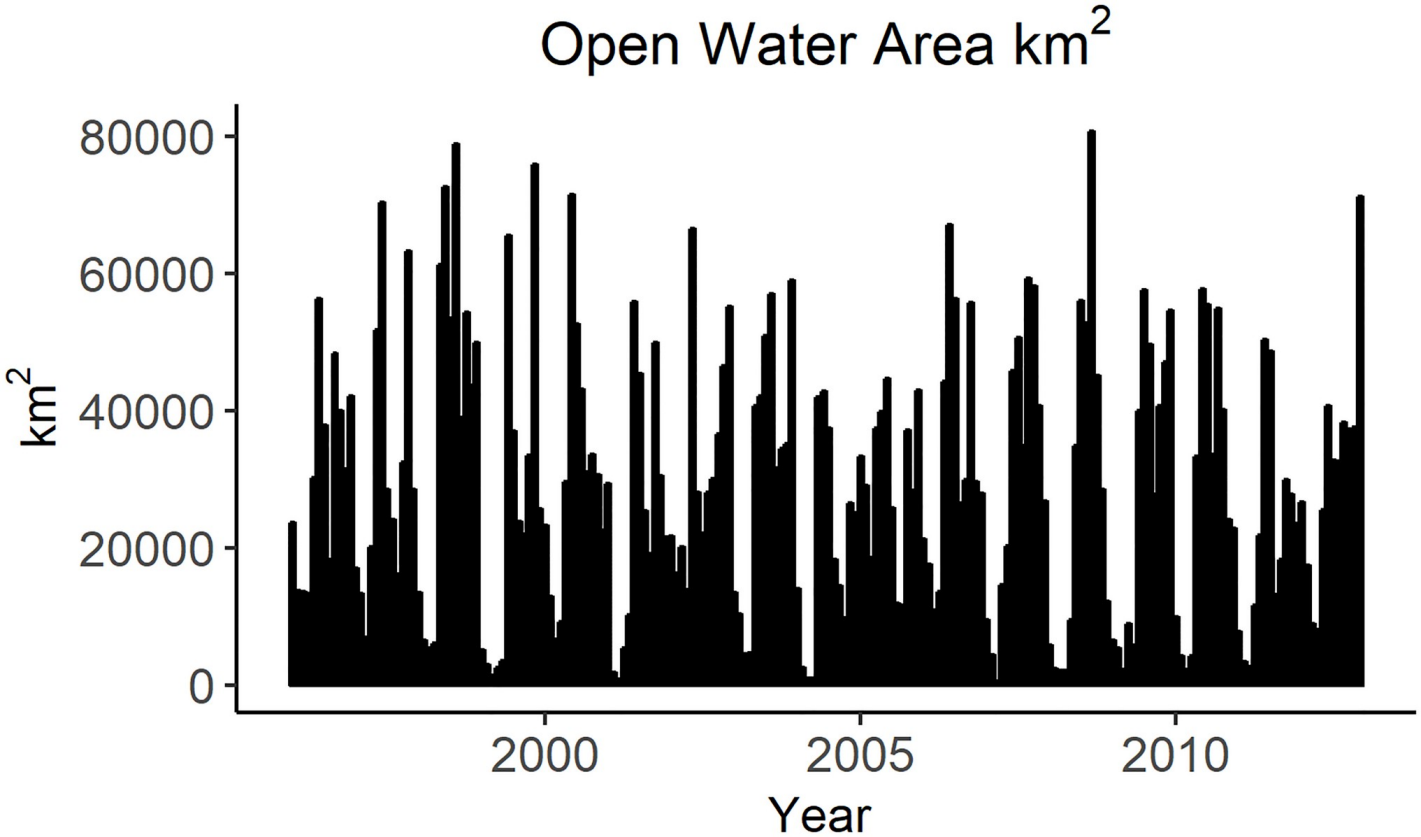
Open water area as documented by the Palmer LTER.

While some Antarctic species thrive in icy conditions, other species have increased success in open water. Gentoo penguin populations have been increasing as the amount of sea ice in the region has declined [17, 57]. Similarly, Antarctic fur seals are pelagic predators that tend to aggregate at the ice edge or in open water to forage [64, 65]. The average monthly open water area as described in the Palmer LTER data [55] was used as a multiplier to force foraging interactions for these pelagic species (Fig 4).

**Fig 5:**
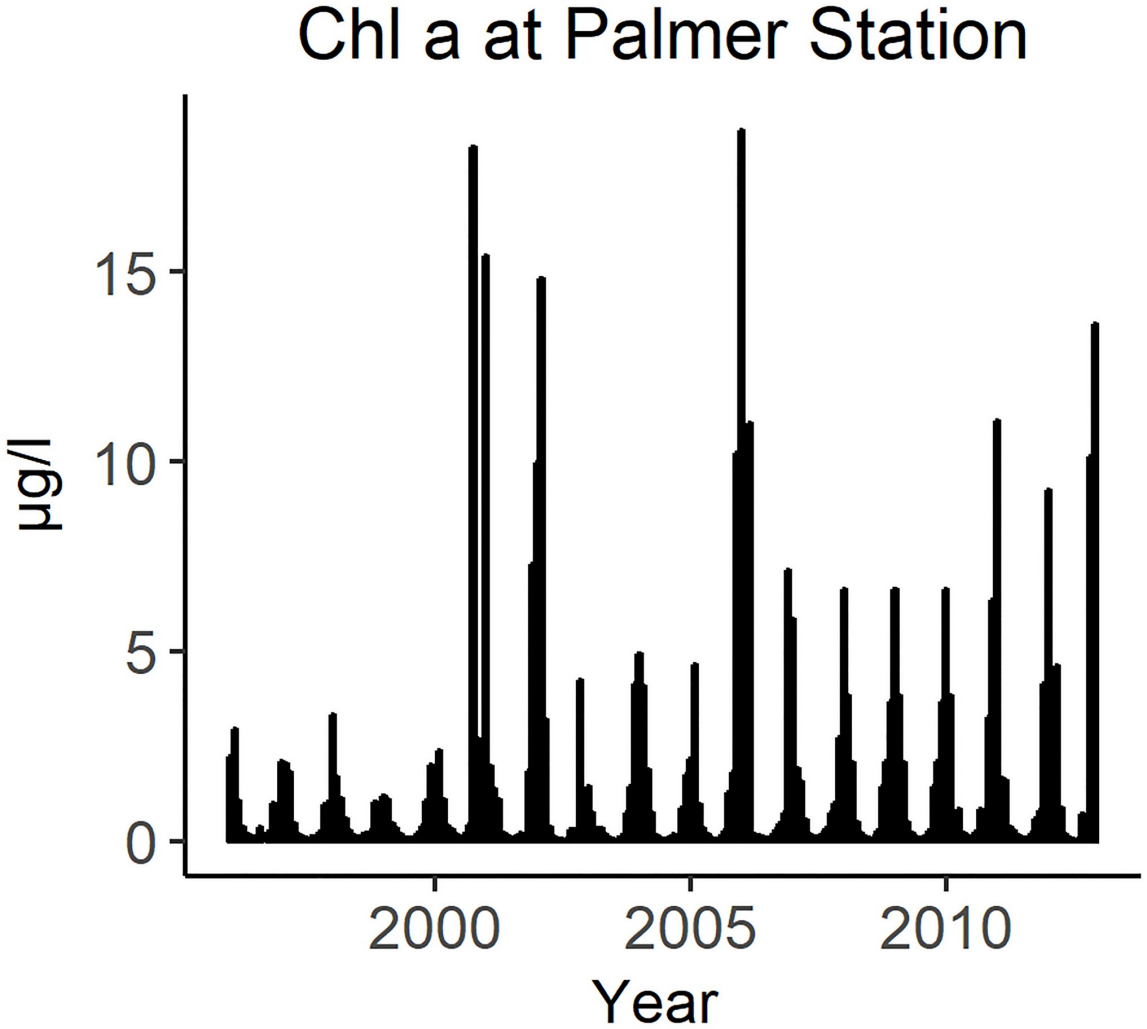
Monthly chlorophyll *a* concentration documented by the Palmer LTER.

The Palmer Long Term Ecological Research (LTER) program has provided weekly measurements of chlorophyll *a* at Palmer Station since 1995 [66]. This is the only year-round chlorophyll time series available for the region during the years 1996–2012; satellite imagery is obscured by clouds during the austral winter [32]. Due to equipment failures, there are several months of missing data in the Palmer LTER chlorophyll *a* time-series. The long-term monthly average was used to approximate the missing months of data. The chlorophyll *a* forcing function (Fig 5) was applied to the primary producers, using the built-in Ecosim multiplier of production rate. This caused primary production in the model to cycle with empirical observations.

**Fig 6:**
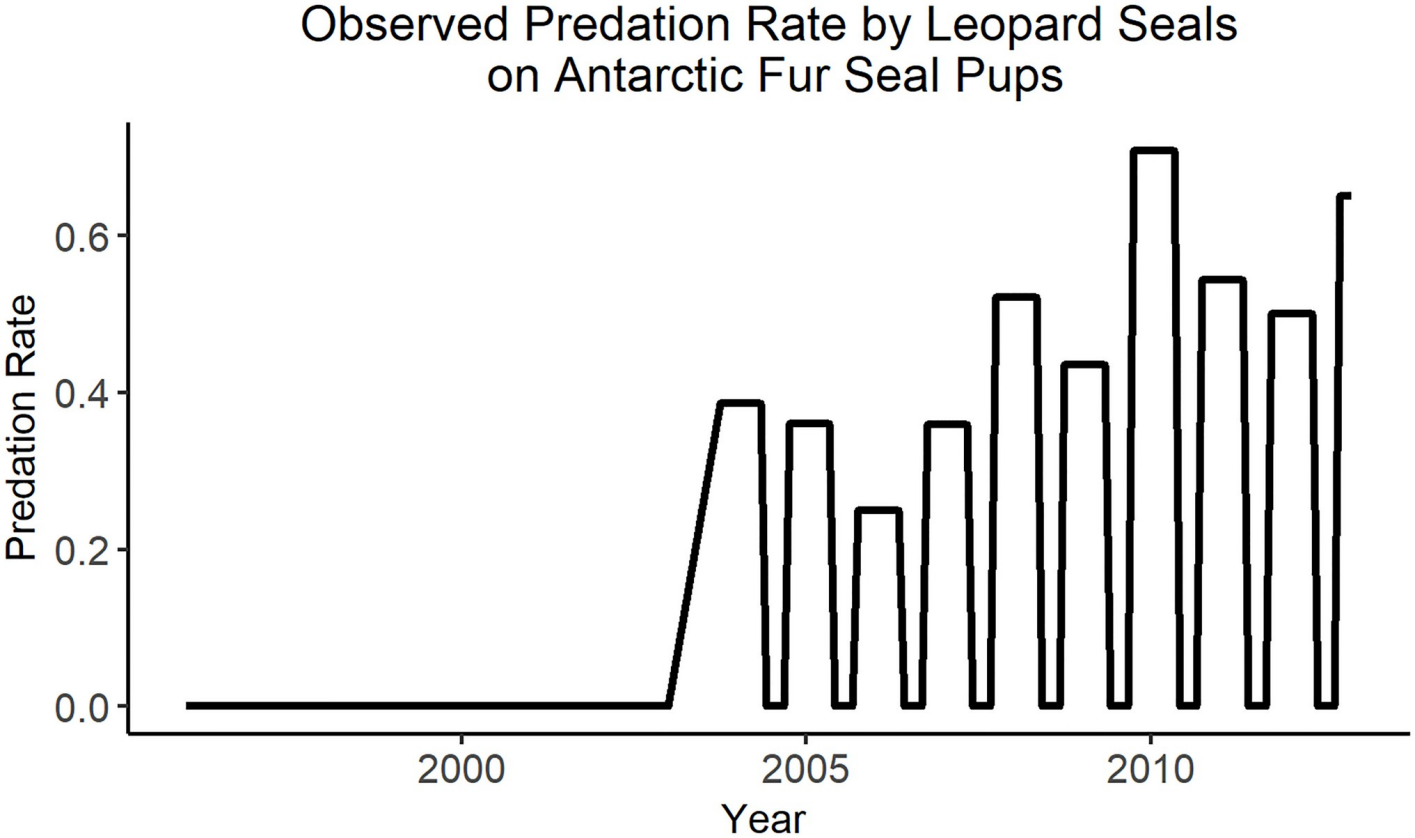
Observed leopard seal predation rate on Antarctic fur seal pups. Figure recreated from Goebel and Reiss [53].

Goebel and Reiss [53] provide a time series (Fig 6) of observed leopard seal predation on Antarctic fur seal pups. These data were used solely to force the trophic interaction between leopard seals and Antarctic fur seals. Goebel and Reiss [53] estimate a single predation rate per Antarctic season. The annual value was repeated for October through May (the season when it was probable to have pups at Cape Shirreff) and zero was assigned to June through September (the months when pups were highly unlikely to be on the beach). Significant leopard seal predation on fur seal pups was first recorded in October of 2003. A value of zero was used from the start of the time series until 2003 [53].

#### Calibration of the model

To begin the calibration process, the model was initially run with only the chlorophyll *a* time series forcing primary producer groups. In this case, the model failed to recreate the documented trends of biomass for all species for which time series were available, except for *C*. *gunnari*. Forcing functions were then applied to specific predator-prey interactions to influence the prey’s vulnerability to predation, the predator’s search rate, or the size of the area where prey are vunerable to predation (i.e., the foraging arena). This type of forcing was applied in Ecosim without the use of response curves. Choices of which time series were applied as forcing functions to each predator-prey interaction were influenced by hunting strategies documented in the diet studies used to build the diet matrix. For example, gentoo penguins forage in near shore environments [67] and have been increasing in abundance as seaice has declined [17]. This suggests that gentoo penguins are more successful foragers in open water conditions. Thus we used the open water environmental driver to force predator-prey interactions between gentoo penguins and on-shelf fish and krill. This application made those prey items more vulnerable to gentoo predation where open water was more prevalent.

Model fit was measured by sums of the squared differences (SS) between simulation results and biomass time series for the eight monitored species, where a smaller SS indicates a better fit. If applying a forcing function to a specific predator prey interaction did not improve the fit, or otherwise help the model recreate trends documented in the time series, that function was no longer applied to that foraging interaction. To improve model fit for large krill and *G*. *gibberifrons*, we used our sea-ice index to directly drive the biomasses of both species. We evaluated two curves for each species and retained the curve that resulted in the lowest total sum of squares. Once the model was able to recreate the trends of biomass as documented in the time-series data, and each group-specifc SS had been minimized, the model was considered calibrated.

We used the Monte Carlo (MC) routine provided within EwE [20] to assess model sensitivity. The MC routine randomly selects initial values of the input parameters (Biomass, P/B, and EE) for all model groups using a coefficient of variation (C.V.) of 0.1 and computes the total sum of squares using these new input values. The degree of difference in the sum of squares between the user-specified model, and randomly selected runs is used to infer sensitivity to small changes in input parameters. We ran 200 MC simulation trials to assess sensitivity.

## Results

### Ecopath

After adjusting the diet matrix (Table 1), the Ecopath model balanced (Table 2).

**Table 1:** Final diet matrix.

Model Group	Prey
**Killer Whale**	3% Leopard Seals, 46.5% Weddell Seals, 36.5% Crabeaters Seals, 1% Elephant Seals, 1% Blue Whales, 1% Fin Whales, 1% Minke Whales, 1% Humpback Whales, <1% Emperor Penguins, <1% Gentoo Penguins, 2% Chinstrap Penguins, <1% Adélie Penguins, 3% Myctophid fish, 2% On-shelf Fish, <1% *N*. *rossii*, 1% *G*. *gibberifrons*
**Leopard Seal**	<1% Antarctic Fur Seals, <1% Gentoo Penguins, 3% Chinstrap Penguins, 7.8% Cephalopods, 4% Myctophids, 15% *G*. *gibberifrons*, 70% Large Krill
**Weddell Seal**	8% Cephalopods, 5% Myctophids, 60% On-shelf Fish, 22% *G*. *gibberifrons*, 5% Benthic Invertebrates
**Crabeater Seal**	7.5% Cephalopods, 7.5% Myctophids, 7% On-shelf Fish, 78% Large Krill
**Antarctic Fur Seal**	1% Gentoo Penguins, 3% Chinstrap Penguins, <1% Adélie Penguins, <1% Macaroni Penguins, 5.4% Cephalopods, 20% Myctophids, 20% On-shelf Fish, 50% Large Krill
**S Elephant Seal**	60% Cephalopods, 10% Myctophids, 14% On-shelf fish, 10% N. rosii, 6% *G*. *gibberifrons*
**Sperm Whale**	85% Cephalopods, <1% Myctophids, 4.5% On-shelf Fish, 10% Benthic Invertebrates
**Blue Whale**	61% Large Krill, 20% Other Euphausiids, 19% Macrozooplankton
**Fin Whale**	1.5% Myctophids, 1.5% On-shelf Fish, 71% Large Krill, 12% Other Euphausiids, 1% Mesozooplankton, 13% Macrozooplankton
**Minke Whales**	1% Myctophids, 1% On-shelf fish, 76% Large Krill, 11% Other euphausiids, 11% Macrozooplankton
**Humpback Whale**	6% Cephalopods, 4% Myctophids, 4% On-shelf Fish, 76% Large Krill, 1.5% Mesozooplankton, 8.5% Macrozooplankton
**Emperor Penguin**	10% Cephalopods, 38% On-shelf Fish, 52% Large Krill
**Gentoo Penguin**	10% Myctophids, 10% On-shelf-fish, 80% Large Krill
**Chinstrap Penguin**	2.25% Myctophids, 2.25% On-shelf Fish, 95% Large Krill, <1% Macrozooplankton
**Adélie Penguin**	1.25% Myctophids, <1% *C*. *gunnari*, 1.25% *G*. *gibberifrons*, 96.2% Large Krill, 1.25% Macrozooplankton
**Macaroni Penguin**	1% Cephalopods, 10% Myctophids, 12% On-shelf Fish, 34% Large Krill, 35% Other Euphausiids, 8% Mesozooplankton
**Flying Birds**	46% Cephalopods, 4.3% Myctophids, 8.7% On-shelf Fish, 30% Large Krill, <1% Mesozooplankton, 10.5% Macrozooplankton
**Cephalopods**	2% Myctophids, 2% On-shelf Fish, 21% Benthic invertebrates, 40% Large Krill, 15% Other Euphausiids, 20% Macrozooplankton
**Myctophids**	25% Large Krill, 35% Other Euphausiids, 5% Mesozooplankton, 35% Macrozooplankton
**On-shelf Fish**	5.5% Cephalopods, 2% Myctophids, 1.5% C. gunnari, 1% Salps, 20% Benthic Invertebrates, 25% Large Krill, 13.5% Other Euphausiids, 8.5% Mesozooplankton, 23% Macrozooplankton
***N*. *rossii***	10% Myctophids, 2% Salps, 2% Benthic Invertebrates, 60% Large Krill, 20% Other Euphausiids, 6% Ice algae
***C*. *gunnari***	1% Myctophids, 90% Large Krill, 8% Other Euphausiids, 1% Macrozooplankton
***G*. *gibberifrons***	1% Cephalopods, 2% Myctophids, 17% Salps, 59% Benthic invertebrates, 9% Large Krill, 2% Macrozooplankton, 10% Ice algae
**Salps**	<1% Small Krill, 10.4% Microzooplankton, 3% Mesozooplankton, 41.5% Small phytoplankton, 45% Large Phytoplankton
**Benthic invertebrates**	100% Detritus
**Large Krill (≥24 months)**	10% Mesozooplankton, 50% Large phytoplankton, 10% Ice Algae, 30% Detritus
**Small Krill (< 24 months)**	10% Microzooplankton, 27.5% Small phytoplankton, 27.5% Large phytoplankton, 25% Ice Algae, 10% Detritus
**Other Euphausiids**	20% Mesozooplankton, 60% Large phytoplankton, 20% Detritus
**Microzooplankton**	60% Small phytoplankton, 25% Large phytoplankton, 15% Detritus
**Mesozooplankton**	3% Microzooplankton, 24% Small phytoplankton, 66% Large phytoplankton, 7% Detritus
**Macrozooplankton**	1% Large Krill, 2% Small Krill, 1% Other euphausiids, 50% Mesozooplankton, 10% Small phytoplankton, 21% Large phytoplankton

**Table 2:** Balanced ecopath model.

Model Group	B (t/100km^2^)	P/B	Q/B	EE	Trophic Level
**Killer Whale**	0.75	0.02	1.08	(0.00)	(4.72)
**Leopard Seals**	0.84	0.27	15.17	(0.11)	(3.41)
**Weddell Seal**	8.12	0.08	4.60	(0.584)	(4.16)
**Crabeater Seal**	109.78	0.10	5.95	(0.03)	(3.36)
**Antarctic Fur Seal**	0.10	0.17	9.66	(0.72)	(3.69)
**S Elephant Seal**	0.10	0.21	12.07	(0.37)	(4.23)
**Sperm Whale**	2.84	0.29	16.67	(0.00)	(4.12)
**Blue Whale**	0.72	0.04	2.53	(0.28)	(3.21)
**Fin Whales**	4.28	0.03	2.55	(0.98)**	(3.21)
**Minke Whales**	4.73	0.10	5.65	(0.02)	(3.19)
**Humpback Whale**	8.12	0.04	2.38	(1.0)**	(3.30)
**Emperor Penguin**	0.01	0.19	13.89	(0.00)	(3.67)
**Gentoo Penguins**	0.12	0.22	15.28	(0.95)	(3.34)
**Chinstrap Penguin**	2.14	0.22	15.28	(0.90)	(3.16)
**Adélie Penguin**	0.58	0.12	36.62	(0.12)	(3.14)
**Macaroni Penguin**	0.01	0.11	7.64	(0.79)	(3.45)
**Flying birds**	0.40	0.09	4.89	(0.00)	(3.83)
**Cephalopods**	249.00	3.15	30.29	(0.29)	(3.24)
**Myctophids**	327.00	1.10	10.58	(0.75)	(3.30)
**On-shelf fish**	525.00	0.46	4.42	(0.93)	(3.30)
***N*. *rossi***	13.80	0.29	2.79	(0.03)	(3.18)
***C gunnari***	90.00	0.48	4.62	(0.81)	(3.13)
***G gibberifrons***	120.00	0.46	4.42	(0.19)	(2.98)
**Salps**	16000.00	3.00	12.25	(0.00)	(2.14)
**Benthic invertebrates**	8553.75	0.50	2.19	(0.55)	(2.00)
**Large Krill (≥24 months)**	8126.00	*0.8	3.57	(0.97)	(2.10)
**Small Krill (< 24 months)**	(2893.07)	*0.8	6.51	(0.35)	(2.1)
**Other Euphausiids**	148000.00	1.5	6.70	(0.14)	(2.21)
**Microzooplankton**	2500.00	55	275.00	(0.22)	(2)
**Mesozooplankton**	13000.00	4.81	19.63	(0.71	(2.03)
**Macrozooplankton**	3500.00	2.5	8.93	(0.37)	(2.56)
**Small phytoplankton**	(15023.17)	75		0.5	(1.00)
**Large phytoplankton**	(13712.00)	75		0.5	(1.00)
**Ice algae**	(306.67)	50		0.5	(1.00)
**Detritus**	577.00			(0.11)	(1.00)

Values in parentheses were calculated by the model. Values marked with an asterisk (*) are Z values for the multi stanza description of krill.

EE values marked with two asterisks (**) include a biomass accumulation term. All values have been rounded to 2 decimal places. Biomass values have been multiplied by 100 km for ease of presentation.

### Ecosim

After applying forcing functions and drivers, and fitting the model to observations, the model was successfully calibrated for the seventeen-year period 1996–2012. The application of forcing functions to specific predator-prey interactions improved the fit of the model by reducing sum of squares differences between model results and time-series data. A list of forcing function applications retained in the final model is presented in Table 3. While many alternative applications of forcing functions were evaluated, we retained only those that reduced the total SS, with two notable exceptions. The final model includes open water forcing of the interaction between on-shelf fish and *C*. *gunnari* and the interaction between *C*. *gunnari* and other euphausiids because they are necessary for the model to recreate the increase in *C*. *gunnari* biomass documented in time-series observations of this species [63]. To assess the impact of each forcing function application on the total SS, we singularly removed each forcing function application and noted the difference in total SS.

**Table 3:** Forcing function applications retained in the final model to influence predator-prey interactions.

Predator	Prey Forcing
**Killer Whale**	Gentoo penguin vulnerability increases with open water (0.62)
**Leopard Seal**	Antarctic fur seal vulnerability increases with open water and observed predation rate (2.16); Chinstrap penguins vulnerability increases with sea-ice index (0.35); Myctophids arena area increases with sea-ice index (0.15)
**Antarctic Fur Seal**	Cephalopods vulnerability and arena area increase with open water (0.09); On-shelf fish vulnerability and arena area increase with open water (0.68); Search rate for Large krill increases with sea-ice index (0.1)
**Gentoo Penguin**	On-shelf-fish vulnerability increases with open water (0.69); Large krill vulnerability increase with open water (0.43)
**Chinstrap Penguin**	Myctophids vulnerability and arena area increase with sea-ice index (0.09); On-shelf fish vulnerability and arena area increase with sea-ice index (0.1); Large Krill vulnerability and arena area increase with sea-ice index (1.33); Macrozooplankton vulnerability and arena area increase with sea-ice index (0.02)
**On-shelf fish**	*C*. *gunnari* arena area increases with open water (-2.91)
***N*. *rossii***	Large krill vulnerability increases with open water (2.22); Other euphausiids vulnerability increases with open water (8.32)
***C gunnari***	Other euphausiids vulnerability increases with open water (-4.06)
**Large Krill (≥24 months)**	Mesozooplankton vulnerability increases with sea-ice index (0.04); Large phytoplankton vulnerability and arena area increase with chlorophyll-*a* (3.8); Ice algae vulnerability and arena area increase with chlorophyll-*a* (1.48)
**Small Krill (<24 months)**	Small phytoplankton vulnerability and arena area increase with sea-ice index (0.17); Large phytoplankton arena area increases with chlorophyll-*a* (0.82); Ice algae vulnerability and arena area increase with sea-ice index (1.09)

The predator column indicates the impacted predator of the predator-prey interaction. The Prey Forcing column indicates the prey item and which forcing function was applied. The values in parentheses indicate the change in total SS when that forcing was removed. If a predator does not appear in the table, interactions with its prey are not forced in the final model.

**Fig 7:**
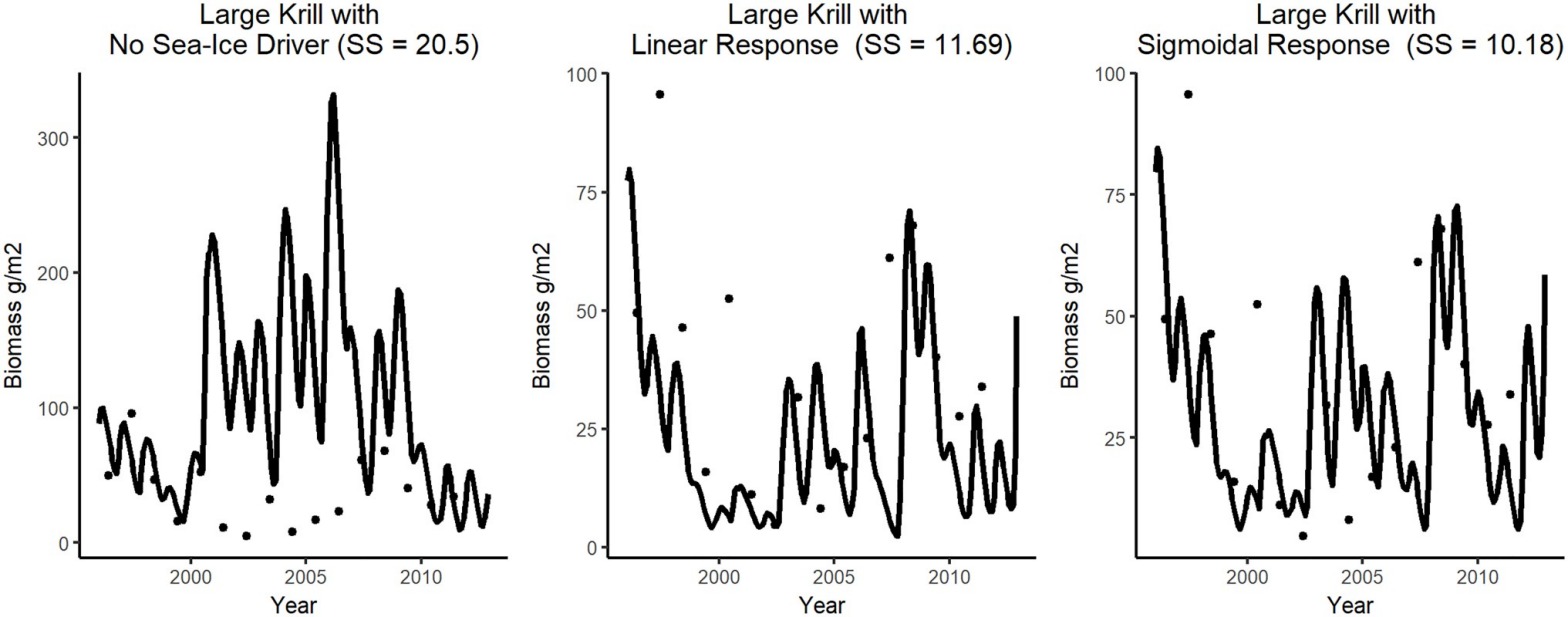
Evaluation of response curves for krill. Comparison of model fits (by group-specific SS) for krill when no curve was applied (a), the linear functional response curve was applied (b), and the sigmoidal functional response curve was applied(c). Curves are displayed in Fig 3 In all panels the dots represent the observed data and the lines represent model results. Note that the y-scale in panel a is significantly larger than the other two panels.

We used the sea-ice index to drive the biomasses of krill and *G*. *gibberifrons*. The linear response curve improved the group-specific SS for Large Krill from 20.5 to 11.69 (Fig 7). Applying a sigmoidal response curve with a steep positive response further improved the group-specific SS of Large Krill to 10.18 and coincidently made slight improvements to the fits for Adélie penguin, chinstrap penguin, gentoo penguin, *C*. *gunnari* and *G*. *gibberifrons*. The total SS for the model, was approximately four less using the sigmoidal response curve than when using the linear response curve. We retained the sigmoidal response curve in the model. The model fit for krill was further improved after the fit for *G*. *gibberifrons* improved. Applying a linear curve to drive *G*. *gibberifrons*, changed the trajectory of the model results to align with the documented decline in the biomass of this species and reduced the group-specific SS from 9.321 to an SS of 0.621. Applying a normal response curve to drive *G*. *gibberifrons* decreased model performance, with the group-specific SS rising to 20.20. We retained the linear response curve in the model.

**Fig 8:**
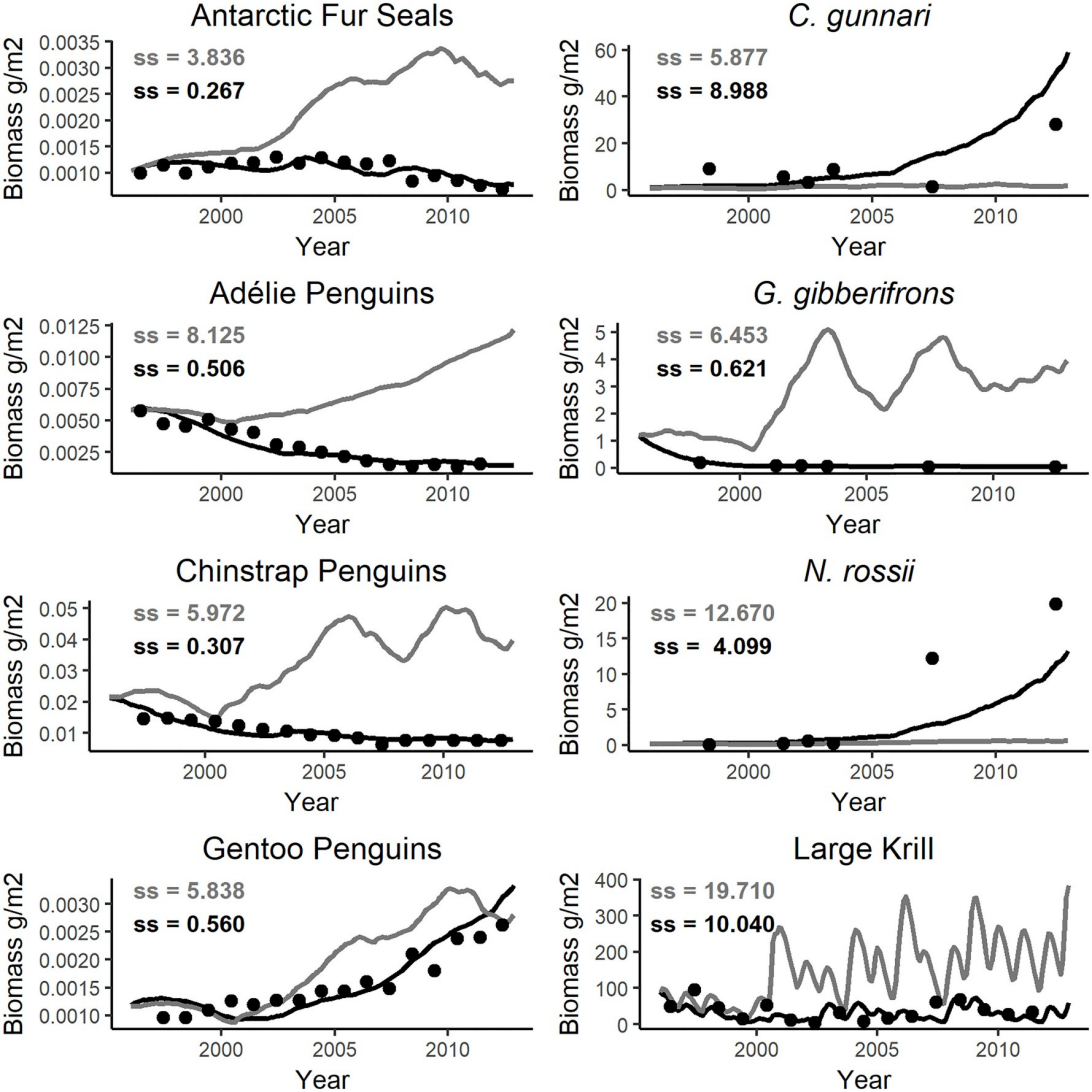
Results of Ecosim simulations. Biomass time series are plotted as black points. The relative biomass results from the model are plotted as lines. Simulations without sea-ice forcing are shown in grey; simulations with sea-ice forcing are shown in black. The group-specific sum of squares (SS) difference between simulation results and observed data are shown for each species.

The total SS for the model without forcing was 70.77 and simulation results did not fit observed trends in biomass (Fig 8). After sea-ice forcing of predator-prey interactions and driving the biomasses of krill and *G*. *gibberifrons*, the group-specific SS for krill alone decreased nearly 50% from 19.71 to 10.04. The total SS dropped to 25.39, and the simulation results better fitted the observed data (Fig 8; model results from the final fitted model for the 27 uncalibrated species are presented in S7 File). Although the total SS decreased considerably, the fit for *C*. *gunnari* worsened but its approximation of the patterns documented in the time series for this species improved (Fig 8). Sums of squares difference for each species group ranged from less than 0.5 to 10.04 in the final fitted model. Species for which yearly data points were available, and which demonstrated an obvious trend in the biomass, had the smallest group-specific SS values. The largest group-specific SS (10.04) was associated with large krill, which has high variability in the time-series data and lacks an obvious trend in biomass. Krill data were entered into the model without any smoothing because krill biomass is known to be highly variable both temporally and spatially [12, 13]. While the model was not able to recreate all the variability evident in the krill dataset, the simulation result was a reasonable approximation of krill’s temporal dynamics in Statistical Subarea 48.1.

**Fig 9:**
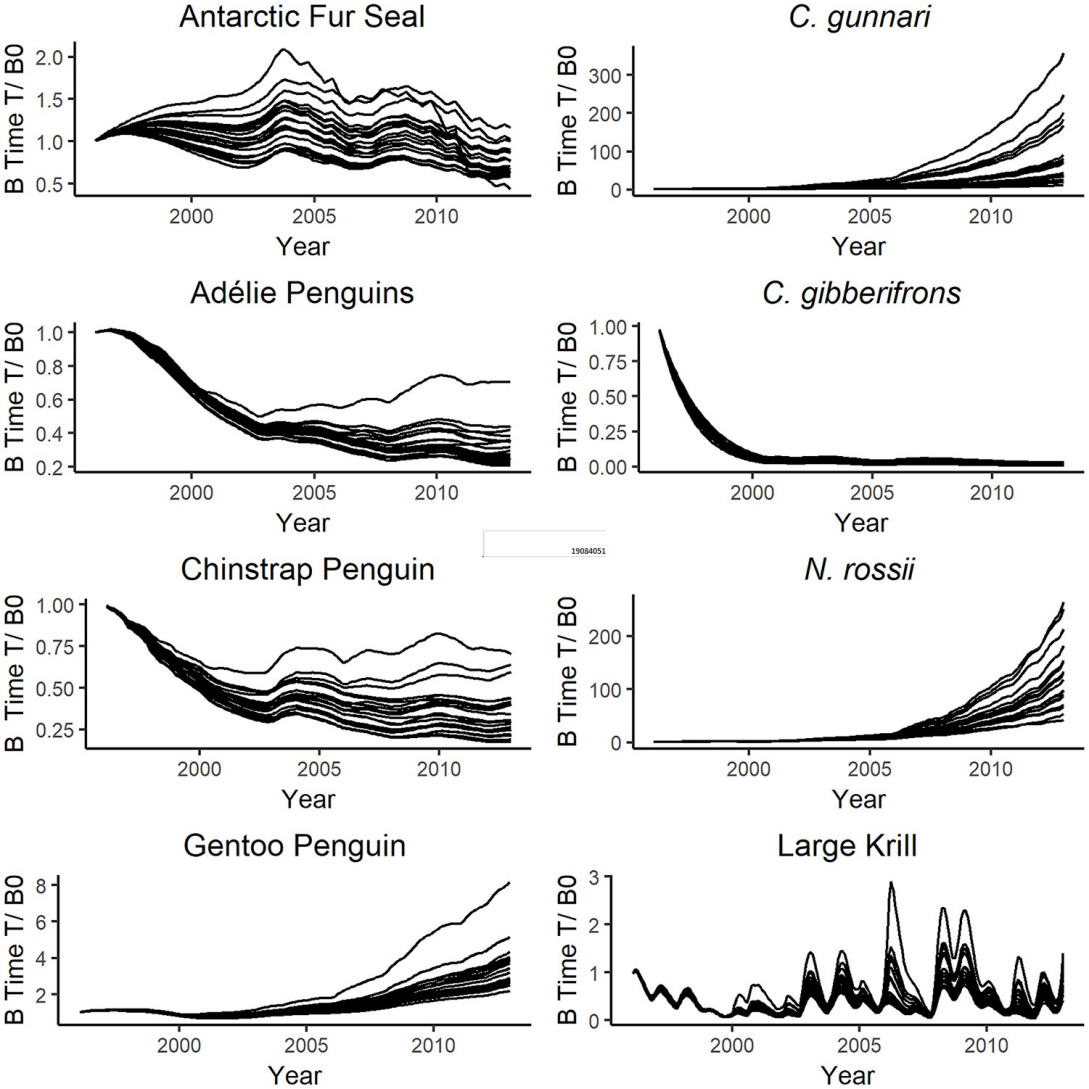
Results of twenty randomly selected Monte Carlo trials. Each line represents the relative biomass trajectory of that species over the course of a single trial. Note that the starting value for all species is one, and model results is relative to that value. Also Note that the y-axis scales for *N*. *rossii* and *C*. *gunnari* are two to three orders of magnitude larger than the scales for the other species, indicating much higher sensitivity and uncertainty for these two species.

The MC sensitivity analysis yielded 200 simulations that produced balanced models. The total SS for each simulation varied between 21.85 and 38.17. The groups that exhibit the highest variability and contributed most to the SS, and thus were the most sensitive to the input parameters, were the two fish species *N*. *rossii* and *C*. *gunnari* (Fig 9). The “best” MC simulation identified a slightly better total SS than that achieved during the model calibration process. This better fit was achieved on runs where the adjusted input parameters resulted in better fitting simulations for *C*. *gunnari*. Among various differences in biomasses and rates, the best fitting MC simulation had notably higher biomass for large krill (9402 t/100 km^2^), and salps (17458 t/100km^2^) and lower biomass for myctophids (276 t/100 km^2^). We chose the purposefully calibrated model over the best MC simulation because the biomass densities of krill and salps in the former model were more consistent with values found in the literature and better supported by field observations. The best fitting MC simulation used parameter estimates that we consider to be less plausible in reality.

## Discussion

The model was calibrated and successfully recreated observed trends in the biomasses of key monitored species. The calibration of the model represents a significant advancement over the previously published, uncalibrated EwE models for the region [see: 7, 23, 24]. The decrease in total SS by 65% (relative to a balanced but uncalibrated model) highlighted the potential importance of the sea-ice regime in structuring the marine ecosystem of Statistical Subarea 48.1. This finding agrees with long-term ecological studies in the area [1, 2].

The Monte Carlo sensitivity analysis revealed that two fishes (*C*. *gunnari* and *N*. *rossii*) were sensitive to input parameters and were responsible for the greatest variability between runs. This result was unsurprising for four key reasons. First, population-dynamics and diet data for these fishes s are scarce [9, 18]; second, there are large gaps in the biomass time series; third, the group-specific SS associated with *C*. *gunnari* in the fitted model was large compared to other groups; and fourth, small changes in the biomass of other modeled groups, specifically on-shelf fish, could have a large impact on C. gunnari (S8 File, trophic impact analysis). The sensitivity analysis indicated that a better fit could be achieved if the starting biomasses of large krill and salps were both increased, but the levels to which these biomasses would need to be increased do not seem realistic in our view.

### Ecological implications of model choices for the 1996–2012 WAP model

Our model builds on previous Ecopath models of the region [7, 22–25], and uses many of the same data sources as the previous models to inform choices regarding Ecopath and Ecosim parameters. Yet, our model has some key features which could influence ecological interpretation of the results.

As noted by the earlier models, data describing the total biomass of primary production for the region were not readily available. Heymans et al. [34] highlighted that coastal ecosystems with strong seasonal patterns in primary production typically have EE values less than 0.5. By adopting this EE value, we modelled the WAP as if there was ample primary production. The phytoplankton biomass values estimated by the model were high, but Statistical Subarea 48.1 is a region that generally experiences high chl-*a* concentrations and high krill growth rates [68], and high sedimentation rates in the summer [69]. Collectively this indicates that there may be ample food for krill and that setting the EE value to 0.5 was reasonable.

Our model included a larger biomass of krill than most of the earlier models. The increased biomass of krill resulted from using the NOAA-AMLR acoustic time series of krill density (https://swfsc.noaa.gov/AERD-Data/), to both set the biomass in Ecopath and serve as the reference time series used for calibration. The NOAA-AMLR study area may encompass a particularly krill rich region, as densities reported by NOAA-AMLR were higher than the average densities reported in a concurrent synoptic survey for entire the region [70]. With the exception of Cornejo-Donoso and Antezana [22], the previously published models adopted krill biomasses that were more consistent with the average values presented in the synoptic survey [70]. Using a higher biomass in our model could imply that krill predators were less food limited than in the earlier studies. However, that comparison is challenging to make across models. The Ecotrophic Efficiency (EE) for krill in the present study was 0.97, indicating that roughly 97% of the available krill were consumed by predators and caught by the fishery. Cornejo-Donoso and Antezana [22] and Erfran and Pitcher [25] set the EE for krill in their models to 0.95 and allowed their models to estimate the biomass of krill needed to satisfy predators at that level of consumption. The results were very different; the Cornejo-Donoso and Antezana [22] model estimated a biomass of approximately 105 t/km^2^ and Erfran and Pitcher [25]estimated approximately 27 t/km^2^. Suprenand and Ainsworth [23] and Hoover et al. [24], used low biomasses of adult krill (approximately 9 t/km^2^) and also estimated low EEs, both of which were less than 0.8. Collectively, the existing Ecopath models for the region, seem to portray the possibility of food limitations for krill predators quite differently. Such conflicting information may not be helpful to marine resource managers. Our estimate that 97% of krill is either consumed by predators or caught by the fishery is consistent with work by Trivelpiece et al. [54] and Hinke et al. [71] which suggested that in areas where the fishery is active krill predators may be limited by krill availability. Our model used actual field data to describe krill biomass and was calibrated to time-series data for both krill and krill predators. Therefore, we suggest that our model may better suited than the earlier Ecopath models for examining trends in krill biomass and the potential of food limitation for krill predators.

Our model included a large biomass of salps; salp biomass was roughly twice the size of the biomass of large krill. This was a significant departure from the previously published models where salp biomass was well below the biomass of krill [7, 22–25]. The high biomass of salps in this model was informed by the work of Loeb and Santora [72] who found that, in the NOAA-AMLR study area, salp abundance was highly variable but could be as much as six times greater than that of krill. Additionally, Atkinson et al. [16] reported that the Antarctic Peninsula region experienced at least a two-fold increase in salps and a similar decline in krill during the 1990s and early 2000s. Salps rarely appear as a prey item in diet studies this may possibly reflect low consumption of salps or the fact that once consumed, salp tissue because unrecognizable more quickly than the remains of prey items that contain hard body parts. We therefore estimate the EE of salps to be very low. As a result, in our model, much of the primary production was consumed by salps, rather than krill. Primary production was not transferred to higher trophic levels or available for krill to consume. Salp biomass is highly variable and responsive to environmental conditions [72]. If warming and sea-ice loss continue in the region, salp biomass might be expected to increase[16] and consume primary production that krill could have consumed. In setting the EE for primary producers to 0.5 we modelled the system as if krill were not food limited. However, in the future, in areas where salp biomass has increased, it seems possible that krill may be food limited.

The calibration process, which involved fitting the model to times series using forcing functions, provided an opportunity to explore environmental impacts on foraging interactions. Two previously published models developed Ecosim scenarios [23, 24], but those models were not calibrated, and the authors did not detail their application of forcing on predator-prey interactions. We note that one of those models, Hoover et al. [24] also attempted to calibrate their model using sea-ice forcing and evaluated use of the Palmer LTER data describing sea-ice extent [55] to build the sea-ice forcing function. The final model developed by Hoover et al. [24] ultimately used different sea-ice data, only applied sea-ice forcing to the ice algae functional group, used sea-ice to drive the model for larval and juvenile krill rather than larger adult krill, and was ultimately unsuccessful at recreating trends in the abundance of penguins. Our model was the first to be successfully calibrated for the region and we are the first set of authors to list the forcing function applications that helped us fit the model to time-series data. As such, our model is a significant improvement over the Hoover et al. [24] model.

The application of forcing functions yielded some unexpected results. While the impacts of removing a single forcing function application were small, each forcing application influenced the others and their effects were collectively quite large. When all forcing functions and drivers were used the total SS decreased by 65%. Adélie penguins were notably absent from the list of predators where forcing was successfully applied. Hinke et al. [56] found that overwinter foraging success and survival of juvenile penguins was impacted by sea-ice conditions, and that when conditions were icier, over winter foraging success and survival were both higher. Yet, applying sea-ice forcing to Adélie penguin foraging interactions did not improve the model fit. However, model fit was improved by driving large krill, the main prey of Adélie penguins [73] with our sea-ice index. This implied that krill biomass, rather than specific foraging conditions, affected Adélie penguin biomass, a finding consistent with the work of Trivelpiece et al. [54]. We did not expect myctophid vulnerability to predation by chinstrap penguins to increase with the sea-ice index. The foraging arena describes the area where predator and prey are likely to overlap and where prey will be vulnerable to predation [20]. It may be counter intuitive that the foraging arena shared by chinstrap penguins and myctophids would increase in icier conditions. However, this could imply that in icy conditions chinstrap penguins were pushed offshore and into areas where they were more likely to overlap with myctophids. The effect of this forcing was quite small (0.09 reduction of total SS), and it may simply represent fitting to noise in the data. A more influential forcing was applied to the interaction between chinstrap penguins and large krill, where krill vulnerability to predation and foraging arena area increased with the sea-ice index. This forcing reduced the total SS by 1.33, and had the second largest positive impact on total SS. It was also consistent with the findings from Hinke et al. [57] that chinstrap penguins experience greater foraging success and survivorship in icier conditions and from Trivelpiece et al. [54] that krill availability influences predator success. The physical forcing function that had the largest positive effect on model fit was particularly obvious. Ice algae were more vulnerable to consumption by large krill and shared a larger foraging arena with krill when chlorophyll *a* concentration was high (1.48 reduction in total SS).

Two forcing function applications that increased the total SS and worsened model fit were retained in the model. Both functions related to *C*. *gunnari*, and impacted interactions with its other euphausiid prey (2.91 increase in SS) and with its on-shelf fish predators (4.06 increase in SS). Without applying forcing to these interactions, the model was not able to recreate the increase in biomass of *C*. *gunnari* as documented in the time-series data [63]. Systematic surveys have yet to be undertaken since those submitted by Kock and Jones [63], but there is no indication that the most recent estimates are erroneously high. Therefore, it seemed prudent to force the model to recreate an increase in the biomass of this fish, despite worsening fits to other time series. The MC simulations illustrated that the model was particularly sensitive to inputs for *C*. *gunnari*. Given the gaps in the timeseries data, the relatively high uncertainty associated with the Ecopath input parameters, and the demonstrated model sensitivity to the input parameters, our results *for C*. *gunnari* should be viewed with some skepticism.

Our model was designed to investigate the effects of sea ice on biomass. Measures of the sea-ice regime were the primary environmental drivers considered in this study, and the dynamics of roughly 25% of the predator groups in the model were tied to changes in the sea-ice regime through application of various forcing functions. Unsurprisingly, biomass projected by the model thus reflects changes in the sea-ice index. Over the course of the calibration period, the sea-ice index generally declined, except for in 2005 and 2006. The time-series data describing abundance show declines over the same period for five out of the eight species. Two of these species, Antarctic fur seals and Antarctic krill increased in biomass during 2007 and these increases were moderately reflected in the model results. While using the sea-ice index to drive the model for krill significantly improved the total SS and allowed the model to adequately fit the observed time series of krill biomass, the model was not able to recreate all the peaks in the time series. Some factor that is not currently captured by the model is likely influencing krill population dynamics. The three species that increased both in the model results and in the real world, had positive responses to more open water conditions.

### Sea-ice in dynamic simulations

Current climate models indicate that sea-ice loss will continue [74]. In the future, successful management of marine living resources around the WAP could depend on understanding and predicting how species might respond to changes in the sea-ice regime. The simulations presented here investigated the role of temporal sea-ice dynamics as one possible mechanism influencing biomass. This was accomplished by using sea ice to force predation interactions and drive the model for large krill and the fish *G*. *gibberifrons*. Including the sea-ice regime allowed the model to recreate documented biomass trends and improved the fit by decreasing the total SS approximately 65%. While the model now adequately recreates documented changes in biomasses of the eight monitored species, it does not capture all the variability in available time-series data. Some factor not currently included in the model likely has a notable impact on the population dynamics of krill and krill predators. The results of our simulations are consistent with previous studies [2] and indicate that the role of sea-ice in structuring the WAP marine ecosystem may be both central and complex.

The sea-ice index used in our model was a normalized, relative measure of annual minimum sea-ice area. Previous studies used the winter maximum sea-ice to model recruitment and survival of krill dependent predators [56, 57]. Summer is the breeding season for monitored krill predators such as penguins [75] and Antarctic fur seals [3]. These species are central place foragers and are required to return to their colonies to feed their young during the breeding season. Summer environmental conditions, including the interaction of sea-ice, temperature, and precipitation patterns, have been shown to impact breeding success and penguin population dynamics [76, 77]. While summer sea-ice minima, rather than winter maxima, were used in the present study, our work did not contradict earlier findings. Sea-ice loss is impacted by a positive feedback loop of ice-free waters absorbing more solar radiation and warming faster [2, 58, 59]; summers following colder icier winters are therefore likely to be colder and icier. Hinke et al. [56] focused on the northern part of WAP, where there seemed to be more winter sea-ice variability than what was recorded in the Palmer LTER data set. Here we came to the same conclusion as Hinke et al. [56] that seasonal sea-ice dynamics, whether lagged winter maxima in the north or normalized summer minima in the south, influence krill and penguin abundance.

Krill have been considered ice dependent [16, 78, 79], and the size of the krill population has increased following years of increased winter sea-ice extent [16, 41, 60, 61]. However, previously published studies describe more general response patterns. Here we present a well-fitting curve that describes how krill respond to our sea-ice index. The curve might be useful in other modeling studies that explore how sea-ice dynamics affect the regional ecosystem. We assert that our model can be used to explore how krill might respond to future changes in the sea-ice regime as the area continues to warm. Similarly, we identified a sea-ice response curve for *G*. *gibberifrons* that allowed the model to recreate observed trends in the abundance of this fish. A direct link between this species and sea ice has not previously been noted in the literature. While the sea-ice response curve worked in the model, it may represent effects of other (environmental) drivers that have not been well documented for this species.

Forcada et al. [80] indicated that sea-ice conditions alone were unlikely to affect penguin population dynamics but suggested that ice might affect trophic dynamics. Similarly, Trivelpiece et al. [54] suggested that krill availability, which may be tied to sea-ice conditions, influences predator abundance. Our results support the findings of Forcada et al. [80]. We were only able to recreate historic trends in the biomasses of monitored species when the sea-ice regime was simultaneously used to drive krill biomass and influence predator-prey dynamics for 10 predators. Indeed, sea-ice might influence trophic interactions beyond what was suggested by Forcada et al. [80], who focused on penguins.

### Management applications for the model

The Commission for the Conservation of Antarctic Marine Living Resources (CCAMLR), the international organization responsible for managing Antarctic marine living resources, has long recognized that understanding ecosystem structure and processes is essential to managing a sustainable krill fishery [81]. The CCAMLR makes its fisheries management decisions within a conservation framework that considers both harvested and associated species [81, 82]. The CCAMLR may find the model presented here useful when it considers future management strategies for the krill fishery in Statistical Subarea 48.1. The model was created to purposefully explore the influence of changes in the sea-ice regime. It may be possible to develop and explore future sea ice scenarios to evaluate the potential effects of future changes in the sea-ice regime on biomass. Some species, such as Antarctic krill might be expected to decline with ice loss [16]. Our model could help explore how a decrease in krill biomass might impact the biomass of krill predators and other species less directly connected to krill through the food web. Such a study would complement the work done by Klein et al. [83]. Collectively, work using two models to explore the potential impacts of climate change may be particularly useful to CCAMLR as it seeks to meet its conservation objectives.

Recently, the CCAMLR agreed that creating a representative system of marine protected areas could help to both conserve Antarctic marine biodiversity and aid in the management of sustainable fisheries [84]. The CCAMLR has identified the Western Antarctic Peninsula and Scotia Sea region, which includes Statistical Subarea 48.1, as a priority area for developing an MPA [84]. The CCAMLR has also adopted a framework for the establishment of future MPAs [85]. This framework stipulates that MPAs should be created using the best available science and aim to protect key ecosystem processes, among other protection objectives. Trophic interactions affect biomass and are important ecosystem processes to consider for protection. In the face of sustained warming and continued sea-ice loss [1, 2], it could be useful to consider dynamic trophic interactions when planning an MPA.

Our model is intended to aid in the MPA planning process for the region. The groups in our model include single-species groups for most indicator species in Statistical Subarea 48.1 [30, 31]. These species will likely be important as the Members of CCAMLR set conservation goals during the MPA planning process and develop monitoring and management plans. Our model is calibrated to available time-series data. As such, the food-web model and time-dynamic simulations presented here lay the foundation for developing a spatial model that could explore MPA placement while considering the dynamic sea-ice regime and trophic interactions.

## Supporting information

S1 File Taxa represented by model groups. (PDF)

S2 File Biomass sources. (PDF)

S3 File Production to biomass ratio references. (PDF)

S4 File Assimilation and production efficiency values. (PDF)

S5 File Diet matrix sources and notes. (PDF)

S6 File Time series sources. (PDF)

S7 File Calibrated model results for groups without time series data. (PDF)

S8 File Mixed trophic impact analysis. Black indicates a negative impact, white indicates a positive impact; size of the circle indicates strength of the impact.
(TIF)
