# Supplementary material for: Using sea-ice to calibrate a dynamic trophic model for the Western Antarctic Peninsula
Source: PLoS One. 2019 Apr 2;14(4):e0214814. doi: 10.1371/journal.pone.0214814 (PMC6445414; doi:10.1371/journal.pone.0214814)
Supplement: S1 File — (PDF) [file pone.0214814.s001.pdf]

S1 Table. Taxa Represented by Model Groups

Definitions of Model Groups

Name	Represented taxa
**Killer whale**	*Orcinus orca*
**Leopard Seal**	*Hydrurga leptonyx*
**Weddell Seal**	*Leptonychotes wedellii*
**Crabeater Seal**	*Lobodon carcinophagus*
**Antarctic Fur Seal**	*Arctocephalus gazella*
**S Elephant Seal**	*Mirounga leonina*
**Sperm Whale**	*Physeter macrocephalus*
**Blue Whale**	*Balaenoptera musculus*
**Fin Whale**	*Balaenoptera physalus*
**Minke Whales**	*Balaenoptera bonaerensis* and *B. acutorostrata*
**Humpback whale**	*Megaptera novaeangliae*
**Emperor Penguin**	*Aptenodytes forsteri*
**Gentoo Penguin**	*Pygoscelis papua*
**Chinstrap Penguin**	*Pygoscelis antarcticus*
**Adélie Penguin**	*Pygoscelis adeliae*
**Macaroni Penguin**	Eudyptes chrysolophus
**Flying Birds**	Includes: Southern giant petrel (*Macronectes giganteus*), Antarctic petrel (*Thalassoica antarctica*), south polar skua (*Stercorarius maccormicki*), Wilson's storm petrel (*Oceanites oceanicus*), blue petrel (*Halobaena caerulea*), cape petrel (*Daption capense*), black browed albatross (*Thalassarche melanophris*), white chinned petrel (*Procellaria aequinoctialis*), snow petrel (*Pagodroma nivea*), Antarctic fulmar (*Fulmarus glacialoides*), balck bellied storm petrel (*Fregetta tropica*).
**Cephalopods**	Squids (for example *Moreoteuthis sp.*)
**Myctophids (Off shelf)**	Members of the the Myctophidae family, including *Electrona antarctica*
**On-shelf fish**	Fish species that live on the continental shelf, excluding the three species named below
N. rossii	*Notothenia rossii*
C gunnari	*Champsocephalus gunnar*i
G gibberifrons	*Gobionotothen gibberifrons*
**Salps**	*Salpa thompsoni*
**S1Benthic Invertebrates**	Echinoderms, polychaetes and gastropods
**Large Krill (≥24 months)**	*Euphausia superba* older than 2 years and easily detected by acoustics
**Small Krill (< 24 months)**	*Euphausia superba* younger than 2 years and harder to detect by acoutics
**Other Euphausiids**	*Thysanoessa macrura* and *Euphausia crystallorophias*
**Microzooplankton**	Dinoflagellates, aloricates, oligotrichs, other ciliates, and sarcodines *
**Mesozooplankton**	Calanid Copepods, Mertridia sp, and other simmiliar sized organisims, *
**Macrozooplankton**	Ostracods, Parachaueta sp, hyperiid amphipods, gamirid amphipods *
**Small phytoplankton**	Cryptophytes and nanoflagellates smaller than 20 micrometers *
**Large Phytoplankton**	Diatoms larger than 20 micrometers *
**Ice Algae**	Algae that grow on sea-ice
**S1Detritus**	Detritus

* Indicates same definition as Ballerini et al. [1]

References

1. Ballerini T, Hofmann EE, Ainley DG, Daly K, Marrari M, Ribic CA, et al. Productivity and linkages of the food web of the southern region of the western Antarctic Peninsula continental shelf. Prog Oceanogr. 2014;122(0):10-29. doi: http://dx.doi.org/10.1016/j.pocean.2013.11.007.
