# Supplementary material for: Using sea-ice to calibrate a dynamic trophic model for the Western Antarctic Peninsula
Source: PLoS One. 2019 Apr 2;14(4):e0214814. doi: 10.1371/journal.pone.0214814 (PMC6445414; doi:10.1371/journal.pone.0214814)
Supplement: S2 File — (PDF) [file pone.0214814.s002.pdf]

S2 File. Biomass Sources

Literature sources for biomass data used in the model

Model Group	Biomass Source	Confidence in Estimate	Effective year of Estimate
**Killer Whale**	Branch and Butterworth [1]	Medium	1993-1998
**Leopard Seal**	Forcada et al. [2]	Medium	1999
**Weddell Seal**	Forcada et al. [2]	Medium	1999
**Crabeater Seal**	Forcada et al. [2]	Medium	1999
**Antarctic Fur Seal**	Hucke-Gaete et al. [3]	High	2002
**Southern Elephant Seal**	Carlini et al. [4]	Medium	1996
**Sperm Whale**	Branch and Butterworth [1]	Medium	1998
**Blue Whale**	Branch and Butterworth [1]	Medium	1998
**Fin Whale**	Hedley et al. [5]	Medium	2000
**Minke Whales**	Branch and Butterworth [6]	Medium	2000
**Humpback whales**	Hedley et al. [5]	Medium	2000
**Emperor Penguins**	Trathan et al. [7], Coria and Montalti [8]	High	1998
**Gentoo Penguins**	Hill et al. [9]	High	2002
**Chinstrap Penguin**	Hill et al. [9]	High	2002
**Adélie Penguin**	Hill et al. [9]	High	2002
**Macaroni Penguin**	Woehler [10]	High	1992
**Flying Birds**	Ribic et al. [11]	Low	1995-2002
**Cephalopods**	Hoover et al. [12], Jackson et al. [13]	Low	1996
**Myctophids (Off shelf)**	Hill et al. [9], Pusch et al. [14]	Medium	1996
**On-shelf fish**	Kock and Jones [15]	Low	1998
N. rossii	Kock and Jones [15]	Low	1999
C. gunnari	Kock and Jones [15]	Low	1998
G. gibberifrons	Kock and Jones [15]	Low	1998
**Salps**	Hoover et al. [12], Loeb and Santora [16], Ballerini et al. [17]	Low	2000
**Benthic Invertebrates**	Ballerini et al. [17]	Low	2001
**Large Krill (≥24 months)**	AMLR data available at: https://swfsc.noaa.gov/AERD-Data/	High	1996-2001
**Small Krill (< 24 months)**	EwE calculated from adult biomass	Low	1996-2001
**Other Euphausiids**	Estimated following Ballerini et al. [17]	Low	2001
**Microzooplankton**	Estimated following Ballerini et al. [17]	Low	2001
**Mesozooplankton**	Estimated following Ballerini et al. [17]	Low	2001
**Macrozooplankton**	Estimated following Ballerini et al. [17]	Low	2001
**Detritus**	Estimated following Hoover et al. [12]	Low	1996

Confidence determination was made based on data sources. Those derived from other Ecopath models [12, 17] and studies for which sampling only covered a small portion of the population [15] or occurred in regions outside of the study area [11] were deemed low confidence. Studies which covered the entirety of the population in the region [7, 8], sampled a significant portion of the study area every year of the calibration period (AMLR krill data), or studies that had taken

measures to bound uncertainty associated with estimates [9] were classified as high confidence. Studies intermediate between these states were considered medium confidence.

References

1. Branch TA, Butterworth DS. Estimates of abundance south of 60°S for cetacean species sighted frequently on the 1978/79 to 1997/98 IWC/IDRC-SOWER sighting surveys. Journal of Cetacean Research and Management. 2001;3(3):251-70.

2. Forcada J, Trathan PN, Boveng PL, Boyd IL, Burns JM, Costa DP, et al. Responses of Antarctic pack-ice seals to environmental change and increasing krill fishing. Biological Conservation. 2012;149(1):40-50. doi: 10.1016/j.biocon.2012.02.002. PubMed PMID: WOS:000305723400006.

3. Hucke-Gaete R, Osman LP, Moreno CA, Torres D. Examining natural population growth from near extinction: the case of the Antarctic fur seal at the South Shetlands, Antarctica. Polar Biology. 2004;27(5):304-11. doi: 10.1007/s00300-003-0587-8.

4. Carlini AR, Daneri GA, Marquez MEI, Soave GE, Poljak S. Mass transfer from mothers to pups and mass recovery by mothers during the post-breeding foraging period in southern elephant seals (*Mirounga leonina*) at King George Island. Polar Biology. 1997;18(5):305-10. doi: 10.1007/s003000050192.

5. Hedley S, Reilly S, Borberg J, Holland R, Hewitt R, Watkins J, et al. Modelling whale distribution: a preliminary analysis of data collected on the CCAMLR-IWC Krill Synoptic Survey, 2000. IWC SC/ 53/ E9. 2001.

6. Branch TA, Butterworth DS. Southern Hemisphere minke whales: standardised abundance estimates from the 1978/79 to 1997/98 IDCR-SOWER surveys. Journal of Cetacean Research and Management. 2001;3(2):143-74.

7. Trathan PN, Fretwell PT, Stonehouse B. First recorded loss of an emperor penguin colony in the recent period of Antarctic regional warming: implications for other colonies. PLoS One. 2011;6(2):e14738. doi: 10.1371/journal.pone.0014738.

8. Coria NR, Montalti D. A newly discovered breeding colony of emperor penguins *Aptenodytes forsteri*. Marine Ornithology. 2000;28(2):119-20.

9. Hill SL, Reid K, Thorpe SE, Hinke J, Watters GM. A compilation of parameters for ecosystem dynamics models of the Scotia Sea-Antarctic Peninsula region. CCAMLR Science. 2007;14:1-25. PubMed PMID: WOS:000251043400001.

10. Woehler E. The distribution and abundance of Antarctic and sub-Antarctic Penguins. Scientific Committee on Antarctic Research. 1993;Cambridge, 76 pp.

11. Ribic CA, Ainley DG, Glenn Ford R, Fraser WR, Tynan CT, Woehler EJ. Water masses, ocean fronts, and the structure of Antarctic seabird communities: putting the eastern Bellingshausen Sea in perspective. Deep Sea Research Part II: Topical Studies in Oceanography. 2011;58(13–16):1695-709. doi: http://dx.doi.org/10.1016/j.dsr2.2009.09.017.

12. Hoover C, Pitcher T, Pakhomov E. The Antarctic Peninsula Marine Ecosystem Model and Simulations: 1978- Present. In: Wabnitz CC, Hoover C, editors. From the tropics to the poles: ecosystem models of Hudson Bay, Kaloko-Honokohau, Hawai'i, and the Antarctic Peninsula, Fisheries Centre Research Reports 20(2). Unversity of British Columbia: Fisheries Centre; 2012. p. 108-82.

13. Jackson GD, Finn J, Nicol S. Planktonic cephalopods collected off East Antarctica during the 'BROKE' survey. Deep Sea Research Part I: Oceanographic Research Papers. 2002;49(6):1049-54. doi: http://dx.doi.org/10.1016/S0967-0637(02)00014-6.

14. Pusch C, Hulley PA, Kock KH. Community structure and feeding ecology of mesopelagic fishes in the slope waters of King George Island (South Shetland Islands, Antarctica). Deep Sea Research Part I: Oceanographic Research Papers. 2004;51(11):1685-708. doi: http://dx.doi.org/10.1016/j.dsr.2004.06.008.

15. Kock K-H, Jones CD. Fish stocks in the southern Scotia Arc region—a review and prospects for future research. Reviews in Fisheries Science. 2005;13(2):75-108. doi: 10.1080/10641260590953900.

16. Loeb VJ, Santora JA. Population dynamics of *Salpa thompsoni* near the Antarctic Peninsula: growth rates and interannual variations in reproductive activity (1993–2009). Prog Oceanogr. 2012;96(1):93-107. doi: http://dx.doi.org/10.1016/j.pocean.2011.11.001.

17. Ballerini T, Hofmann EE, Ainley DG, Daly K, Marrari M, Ribic CA, et al. Productivity and linkages of the food web of the southern region of the western Antarctic Peninsula continental shelf. Prog Oceanogr. 2014;122(0):10-29. doi: http://dx.doi.org/10.1016/j.pocean.2013.11.007.
