# Supplementary material for: Using sea-ice to calibrate a dynamic trophic model for the Western Antarctic Peninsula
Source: PLoS One. 2019 Apr 2;14(4):e0214814. doi: 10.1371/journal.pone.0214814 (PMC6445414; doi:10.1371/journal.pone.0214814)
Supplement: S3 File — (PDF) [file pone.0214814.s003.pdf]

S3 File. Production to Biomass Ratio References

Literature sources for production to biomass ratio data

Model Group	P/B Source	Confidence in Estimate
Killer Whale	Brault and Caswell [1], Kuningas et al. [2]	High
Leopard Seal	Jessopp et al. [3]	High
Weddell Seal	Hadley et al. [4]	High
Crabeater Seal	Croxall [5]	High
Antarctic Fur Seal	Schwarz et al. [6]	High
S Elephant Seal	McMahon et al. [7]	High
Sperm Whale	Ralls et al. [8]	High
Blue Whale	Branch et al. [9]	High
Fin Whale	Hill et al. [10]	High
Minke Whales	Zhang et al. [11]	High
Humpback Whale	Barlow and Clapham [12], Buckland [13]	High
Emperor Penguin	Jenouvrier et al. [14]	High
Gentoo Penguin	Hill et al. [10]	High
Chinstrap Penguin	Hill et al. [10]	High
Adélie Penguin	Hinke et al. [15]	High
Macaroni Penguin	Horswill et al. [16]	High
Flying Birds	Jenouvrier et al. [14], Dobson and Jouventin [17], Ainley et al. [18], Weimerskirch et al. [19], Jenouvrier et al. [20]	Low
Cephalopods	Cornejo-Donoso and Antezana [21], [22]	Low
Myctophids (Off shelf)	Hill et al. [10]	Medium
On-shelf fish	Hill et al. [10]	Medium
N. rossii	Kock and Jones [23]	Medium
C. gunnari	Iverson [24]	Medium
G. gibberifrons	Hill et al. [10]	Medium
Salps	Ballerini et al. [22]	Low
Benthic Invertebrates	Cornejo-Donoso and Antezana [21]	Low
Large Krill (≥24 months)	Rosenberg et al. [25], Candy and Kawaguchi [26]	Medium
Small Krill (< 24 months)	Rosenberg et al. [25], Candy and Kawaguchi [26]	Medium
Other Euphausiids	Ballerini et al. [22]	Low
Microzooplankton	Ballerini et al. [22]	Low
Mesozooplankton	Ballerini et al. [22]	Low
Macrozooplankton	Ballerini et al. [22]	Low

Small phytoplankton	Ballerini et al. [22]	Low
Large Phytoplankton	Ballerini et al. [22]	Low
Ice Algae	Ballerini et al. [22]	Low

Confidence was assigned based on study type. Estimates derived from published studies of marine mammal and penguin natural mortality rates were classified as high confidence. Estimates derived from other Ecopath models were classified as low confidence. All other estimates were classified as medium confidence.

References

1. Brault S, Caswell H. Pod-specific demography of killer whales (*Orcinus Orca*). Ecology. 1993;74(5):1444-54. doi: 10.2307/1940073.

2. Kuningas S, Similä T, Hammond PS. Population size, survival and reproductive rates of northern Norwegian killer whales (*Orcinus orca*) in 1986–2003. Journal of the Marine Biological Association of the United Kingdom. 2014;94(06):1277-91. doi: https://doi.org/10.1017/S0025315413000933.

3. Jessopp MJ, Forcada J, Reid K, Trathan PN, Murphy EJ. Winter dispersal of leopard seals (*Hydrurga leptonyx)*: environmental factors influencing demographics and seasonal abundance. Journal of Zoology. 2004;263(3):251-8. doi: 10.1017/S0952836904005102.

4. Hadley GL, Rotella JJ, Garrott RA. Evaluation of reproductive Costs for Weddell seals in Erebus Bay, Antarctica. Journal of Animal Ecology. 2007;76(3):448-58. doi: 10.2307/4539148.

5. Croxall JP. The status and conservation of Antarctic seals and seabirds: a review. Environment International. 1987;13(1):55-70. doi: http://dx.doi.org/10.1016/0160-4120(87)90044-4.

6. Schwarz LK, Goebel ME, Costa DP, Kilpatrick AM. Top-down and bottom-up influences on demographic rates of Antarctic fur seals Arctocephalus gazella. Journal of Animal Ecology. 2013;82(4):903-11. doi: 10.1111/1365-2656.12059.

7. McMahon CR, Burton HR, Bester MN. A demographic comparison of two southern elephant seal populations. Journal of Animal Ecology. 2003;72(1):61-74. doi: 10.2307/3505543.

8. Ralls K, Brownell Jr RL, Ballou J. Differntial mortality by sex and age in mammals, with specific reference to the sper. Report of the International Whaling Commission Special Issue. 1980;2:233-43.

9. Branch TA, Matsuoka K, Miyashita T. Evidence for increase in Antarctic blue whales based on Bayesian modelling Marine Mammal Science. 2004;20(4):726-54. doi: 10.1111/j.1748-7692.2004.tb01190.x.

10. Hill SL, Reid K, Thorpe SE, Hinke J, Watters GM. A compilation of parameters for ecosystem dynamics models of the Scotia Sea-Antarctic Peninsula region. CCAMLR Science. 2007;14:1-25. PubMed PMID: WOS:000251043400001.

11. Zhang CI, Song K-J, Na J-H. Estimation of mortality coefficients and survivorship curves for minke whales (Balaenoptera acutorostrata) in Korean waters. Animal Cells and Systems. 2010;14(4):291-6. doi: 10.1080/19768354.2010.525807.

12. Barlow J, Clapham PJ. A new birth-interval approach to estimating demographic parameters of humpback whales. Ecology. 1997;78(2):535-46. doi: 10.2307/2266028.

13. Buckland ST. Estimation of survival rates from sightings of individually identifiable whales. Report of the International Whaling Commission Special Issue. 1990;12:149-53.

14. Jenouvrier S, Barbraud C, Cazelles B, Weimerskirch H. Modelling population dynamics of seabirds: importance of the effects of climate fluctuations on breeding proportions. Oikos. 2005;108(3):511-22. doi: 10.1111/j.0030-1299.2005.13351.x. PubMed PMID: 15998808.

15. Hinke J, Trivelpiece S, Trivelpiece W. Adélie penguin (*Pygoscelis adeliae*) survival rates and their relationship to environmental indices in the South Shetland Islands, Antarctica. Polar Biology. 2014;37(12):1797-809. doi: 10.1007/s00300-014-1562-2.

16. Horswill C, Matthiopoulos J, Green JA, Meredith MP, Forcada J, Peat H, et al. Survival in macaroni penguins and the relative importance of different drivers: individual traits, predation pressure and environmental variability. Journal of Animal Ecology. 2014;83(5):1057-67. doi: 10.1111/1365-2656.12229.

17. Dobson FS, Jouventin P. The trade-off of reproduction and survival in slow-breeding seabirds. Canadian Journal of Zoology. 2010;88(9):889-99. doi: 10.1139/Z10-054. PubMed PMID: 54085114.

18. Ainley DG, Ribic CA, Wood RC. A Demographic study of the south polar skua *Catharacta maccormicki* at Cape Crozier. Journal of Animal Ecology. 1990;59(1):1-20. doi: 10.2307/5155.

19. Weimerskirch H, Clobert J, Jouventin P. Survival in five southern albatrosses and its relationship with their life history. Journal of Animal Ecology. 1987;56(3):1043-55. doi: 10.2307/4965.

20. Jenouvrier S, Barbraud C, Weimerskirch H. Long-term contrasted responses to climate of two Antarctic seabird species. Ecology. 2005;86(11):2889-903. doi: 10.2307/3450803.

21. Cornejo-Donoso J, Antezana T. Preliminary trophic model of the Antarctic Peninsula Ecosystem (Sub-area CCAMLR 48.1). Ecological Modelling. 2008;218(1–2):1-17. doi: http://dx.doi.org/10.1016/j.ecolmodel.2008.06.011.

22. Ballerini T, Hofmann EE, Ainley DG, Daly K, Marrari M, Ribic CA, et al. Productivity and linkages of the food web of the southern region of the western Antarctic Peninsula continental shelf. Prog Oceanogr. 2014;122(0):10-29. doi: http://dx.doi.org/10.1016/j.pocean.2013.11.007.

23. Kock K-H, Jones CD. Fish stocks in the southern Scotia Arc region—a review and prospects for future research. Reviews in Fisheries Science. 2005;13(2):75-108. doi: 10.1080/10641260590953900.

24. Iverson I. Natural mortality in the mackerel icefish (*Champsocephalus gunnari*) around South Georgia. CCAMLR Science. 1998;5:245-57.

25. Rosenberg AA, Beddington JR, Basson M. Growth and longevity of krill during the first decade of pelagic whaling. Nature. 1986;324(6093):152-4. doi: https://doi.org/10.1038/324152a0.

26. Candy SG, Kawaguchi S. Modelling growth of Antarctic krill. II. Novel approach to describing the growth trajectory. Marine Ecology Progress Series. 2006;306:17-30. doi: 10.3354/meps306017.
