# Supplementary material for: Using sea-ice to calibrate a dynamic trophic model for the Western Antarctic Peninsula
Source: PLoS One. 2019 Apr 2;14(4):e0214814. doi: 10.1371/journal.pone.0214814 (PMC6445414; doi:10.1371/journal.pone.0214814)
Supplement: S4 File — (PDF) [file pone.0214814.s004.pdf]

S4 File. Assimilation and Production Efficiency Values

Assimilation efficiency (AE) and production efficiency (PE).

Model Group	AE source	AE	PE	P/Q	Confidence in Estimate
Killer Whale	Lockyer [1]	0.93	0.02	0.0186	Low
Leopard Seal	Ballerini et al. [2], Townsend et al. [3]	0.89	0.02	0.0178	Low
Weddell Seal	Ballerini et al. [2], Townsend et al. [3]	0.87	0.02	0.0174	Low
Crabeater Seal	Priddle et al. [4]	0.84	0.02	0.0168	Low
Antarctic Fur Seal	Fadely et al. [5]	0.88	0.02	0.0176	Low
S Elephant Seal	Ballerini et al. [2], Townsend et al. [3]	0.87	0.02	0.0174	Low
Sperm Whale	Ballerini et al. [2], Townsend et al. [3]	0.87	0.02	0.0174	Low
Blue Whale	Lockyer [6]	0.79	0.02	0.0158	Low
Fin Whale	Lockyer [1]	0.8	0.02	0.0160	Low
Minke Whales	Lockyer [6], Armstrong and Siegfried [7]	0.84	0.02	0.0168	Low
Humpback Whale	Lockyer [6], Armstrong and Siegfried [7]	0.84	0.02	0.0168	Low
Emperor Penguin	Kirkwood and Robertson [8]	0.7	0.02	0.0140	Low
Gentoo Penguin	Adams et al. [9]	0.72	0.02	0.0144	Low
Chinstrap Penguin	Adams et al. [9]	0.72	0.02	0.0144	Low
Adélie Penguin	Adams et al. [9]	0.72	0.02	0.0142	Low
Macaroni Penguin	Adams et al. [9]	0.72	0.02	0.0144	Low
Flying birds	Ballerini et al. [2], Townsend et al. [3]	0.9	0.02	0.0180	Low
Cephalopods	Ballerini et al. [2], Townsend et al. [3]	0.8	0.13	0.1040	Low
Myctophids (off shelf)	Ballerini et al. [2], Townsend et al. [3]	0.8	0.13	0.1040	Low
On-shelf fish	Ballerini et al. [2], Townsend et al. [3]	0.8	0.13	0.1040	Low
N. rossii	Ballerini et al. [2], Townsend et al. [3]	0.8	0.13	0.1040	Low
C. gunnari	Ballerini et al. [2], Townsend et al. [3]	0.8	0.13	0.1040	Low

G. gibberifrons	Ballerini et al. [2], Townsend et al. [3]	0.8	0.13	0.1040	Low
Salps	Pakhomov et al. [10]	0.7	0.35	0.2450	Low
Benthic invertebrates	Ballerini et al. [2], Townsend et al. [3]	0.58	0.35	0.2030	Low
Large Krill (≥24 months)	Pakhomov et al. [11]	0.64	0.35	0.2240	Low
Small Krill (< 24 months)	Meyer et al. [12]	0.84	0.35	0.2940	Low
Other euphausiids	Pakhomov et al. [11]	0.64	0.35	0.2240	Low
Microzooplankton	Ballerini et al. [2], Townsend et al. [3]	0.8	0.25	0.2000	Low
Mesozooplankton	Ballerini et al. [2], Townsend et al. [3]	0.7	0.35	0.2450	Low
Macrozooplankton	Ballerini et al. [2], Townsend et al. [3]	0.8	0.35	0.2800	Low
Small phytoplankton	Ballerini et al. [2], Townsend et al. [3]	1	1	1	Low
Large phytoplankton	Ballerini et al. [2], Townsend et al. [3]	1	1	1	Low
Ice algae	Ballerini et al. [2], Townsend et al. [3]	1	1	1	Low

All PE values were derived from [3] an use of this highly uncertain data lead to an assessment of low confidence for all P/Q values.

References

1. Lockyer C. All creatures great and smaller: a study in cetacean life history energetics. Journal of the Marine Biological Association of the United Kingdom. 2007;87(04):1035-45.

2. Ballerini T, Hofmann EE, Ainley DG, Daly K, Marrari M, Ribic CA, et al. Productivity and linkages of the food web of the southern region of the western Antarctic Peninsula continental shelf. Prog Oceanogr. 2014;122(0):10-29. doi: http://dx.doi.org/10.1016/j.pocean.2013.11.007.

3. Townsend CR, Begon M, Harper JL. Essentials of Ecology. 2nd ed. Oxford, UK: Blackwell Publishing; 2003.

4. Priddle J, Boyd IL, Whitehouse MJ, Murphy EJ, Croxall JP. Estimates of Southern Ocean primary production—constraints from predator carbon demand and nutrient drawdown. Journal of Marine Systems. 1998;17(1–4):275-88. doi: http://dx.doi.org/10.1016/S0924-7963(98)00043-8.

5. Fadely BS, Worthy GAJ, Costa DP. Assimilation efficiency of northern fur seals determined using dietary manganese. The Journal of Wildlife Management. 1990;54(2):246-51. doi: 10.2307/3809037.

6. Lockyer C. Growth and energy budgets of large baleen whales from the southern hemisphere. In: FAO Advisory Committee on Marine Reseources Research Working Party on Marine Mammals, editor. Mammals in the seas. Fisheries Series. 3: FAO 1981. p. 379-487.

7. Armstrong AJ, Siegfried WR. Consumption of Antarctic krill by minke whales. Antarctic Science. 1991;3(01):13-8. doi: doi:10.1017/S0954102091000044.

8. Kirkwood R, Robertson G. The energy assimilation efficiency of emperor penguins, *Aptenodytes forsteri,* fed a diet of Antarctic krill, *Euphausia superba*. Physiological Zoology. 1997;70(1):27-32. doi: 10.2307/30164280.

9. Adams NJ, Moloney C, Navarro R. Estimated food consumption by penguins at the Prince Edward Islands. Antarctic Science. 1993;5(03):245-52. doi: doi:10.1017/S0954102093000331.

10. Pakhomov EA, Dubischar CD, Strass V, Brichta M, Bathmann UV. The tunicate *Salpa thompsoni* ecology in the Southern Ocean. I. Distribution, biomass, demography and feeding ecophysiology. Mar Biol. 2006;149(3):609-23. doi: 10.1007/s00227-005-0225-9.

11. Pakhomov EA, Perissinotto R, Froneman PW, Miller DGM. Energetics and feeding dynamics of *Euphausia superba* in the South Georgia region during the summer of 1994. Journal of Plankton Research. 1997;19(4):399-423. doi: 10.1093/plankt/19.4.399.

12. Meyer B, Atkinson A, Blume B, Bathmann UV. Feeding and energy budgets of larval Antarctic krill *Euphausia superba* in summer. Marine Ecology Progress Series. 2003;257:167-78. doi: 10.3354/meps257167.
