# Supplementary material for: Using sea-ice to calibrate a dynamic trophic model for the Western Antarctic Peninsula
Source: PLoS One. 2019 Apr 2;14(4):e0214814. doi: 10.1371/journal.pone.0214814 (PMC6445414; doi:10.1371/journal.pone.0214814)
Supplement: S5 File — (PDF) [file pone.0214814.s005.pdf]

S5 File. Diet Matrix Sources and Notes

Table A Literature Sources for diet data.

Model Group	Study Type	Confidence	Diet References
Killer Whale	Predation observations	Low	Pitman and Durban [1], Pitman and Ensor [2]
Leopard Seal	Scat contents, predation observations, diet model	Low	Casaux et al. [3], Forcada et al. [4], Boveng et al. [5]
Weddell Seal	Scat contents	Low	Casaux et al. [6]
Crabeater Seal	Stable isotope	Low	Hückstädt et al. [7]
Antarctic Fur Seal	Scat contents, stable isotope	Low	Casaux et al. [8], Polito and Goebel [9]
S Elephant Seal	Stable isotope	Low	Hückstädt et al. [10]
Sperm Whale	Standardized diet from gut contents	Low	Pauly et al. [11]
Blue Whale	Standardized diet from gut contents, gut contents	Low	Pauly et al. [11], Kawamura [12]
Fin Whale	Standardized diet from gut contents, consumption model, predation observations	Low	Pauly et al. [11], Armstrong and Siegfried [13], Santora et al. [14]
Minke Whales	Standardized diet from gut contents, gut contents consumption model	Low	Pauly et al. [11], Kawamura [12], Armstrong and Siegfried [13]
Humpback Whale	Gut contents, standarized diet from gut contents	Low	Kawamura (1978), Pauly et al. (1998)
Emperor Penguin	Gut contents,	Low	Klages [15], Cherel and Kooyman [16]
Gentoo Penguin	Gut contents, and stable isotope analysis	Low	Miller et al. [17], Polito et al. [18]
Chinstrap Penguin	Gut content and stable isotope, gut contents	Low	Polito et al. [18], Lynnes et al. [19]
Adélie Penguin	Gut contents	Low	Lynnes et al. [19]
Macaroni Penguin	DNA analysis of scat	Low	Deagle et al. [20]
Flying Birds	Literature synthesis, behavorial	Low	Ainley et al. [21], Ainley et al. [22], Malzof and Quintana [23]

	observations, regurgitated pellet analysis		
Cephalopods	Literature synthesis, gut contents, fatty acid analysis	Low	Rodhouse and Nigmatullin [24], Phillips et al. [25], Kozlov [26]
Myctophids (Off shelf)	Gut contents	Low	Pusch et al. [27], Pakhomov et al. [28]
On-shelf fish	Literature synthesis,	Low	La Mesa et al. [29], Barrera-Oro [30]
N. rossii	Gut contents	Low	Casaux and Barrera-Oro [31], Jones et al. [32]
C. gunnari	Gut contents	Low	Main et al. [33], Flores et al. [34], Jones et al. [32]
G. gibberifrons	Literature synthesis, gut contents	Low	Barrera-Oro [30], Casaux and Barrera-Oro [31], Flores et al. [34], Jones et al. [32] Jones et al. [35]
Salps	Gut contents	Low	Pakhomov et al. [36], Perissinotto and A. Pakhomov [37]
Benthic Invertebrates	Model diet	Low	Jarre-Teichmann et al. [38], Ballerini et al. [39]
Large Krill (≥24 months)	Model diet, fatty acid analysis, gut contents	Low	Ballerini et al. [39], Atkinson et al. [40], Atkinson and Snÿder [41], Perissinotto et al. [42]
Small Krill (> 24 months)	Feeding experiments, gut content	Low	Meyer et al. [43], Meyer et al. [44], Töbe et al. [45], Ross et al. [46]
Other Euphausiids	Model diet, prey field observations, stable isotope analysis	Low	Ballerini et al. [39], Hopkins [47], Marrari et al. [48], Donnelly et al. [49], Pers. Comm, J. Walsh, November 2018, unpublished stable isotope data]
Microzooplankton	Model diet, grazing experiements	Low	Ballerini et al. [39], Froneman and Perissinotto [50], Froneman et al. [51]
Mesozooplankton	Model diet, gut contents, grazing experiments	Low	Ballerini et al. [39], Hopkins [47], Pasternak and Schnack-Schiel [52], Swadling et al. [53]
Macrozooplankton	Model diet, gur contents, predation experiments	Low	Ballerini et al. [39], Pakhomov and Froneman [54], Øresland and Ward [55], Pakhomov and Perissinotto [56]

. The study type column provides a gross description of the field methods used to collect diet data. The confidence column relates to the confidence in the percentages of prey items reported in each study being precise for all predators of that model group in the region. Many of the diet studies referenced have small, spatially constrained sample sizes relative to their respective populations and therefore the percentage of mass in the diet was uncertain.

No detailed, regionally specific diet studies exist for the cetacean species included in the model. Killer whale (*Orcinus orca*) is a species that is composed of several ecotypes distinguished, *inter alia*, by diet [1, 2]. At least two distinct ecotypes of killer whale occur in the

WAP. Ecotype A, which feeds on minke whales (*Balaenoptera bonaerensis* and *B. acutorostrata)* and to a lesser extent elephant seals (*Mirounga leonina*), and the more abundant Ecotype B, which feeds on pack ice seals and penguins [1, 2]. Fish-eating killer whales may also occur in the region [2]. The diet presented in our model reflects what whales in the region have been observed eating and is skewed to favor the more abundant Ecotype B. The diets of blue whale (*Balaenoptera musculus*), fin whale (*Balaenoptera physalus*), minke whales (*Balaenoptera bonaerensis* and *B. acutorostrata*) and humpback whale (*Megaptera novaeangliae*) are based on generalized diets presented in Pauly et al (1998). However, the WAP diets are adapted to recognize the importance of krill and other euphausiids as the main planktonic prey items [12-14].

Published diet studies do not adequately describe the percentages of prey items consumed by two pinniped species. A diet study of leopard seal (*Hydrurga leptonyx*) in the region [3] describes a high percentage of krill and a variety of fish in this predator's diet. Observations from elsewhere in the study region indicate that consumption of Antarctic fur seal (*Arctocephalus gazzella*) pups by leopard seals is a significant source of pup mortality [4, 5, 57]. The diet used in the Ecopath model includes consumption of Antarctic fur seals. Three diet studies have been conducted on southern elephant seals in the region, and all three agree that fishes and cephalopods are dietary staples [10, 58, 59]. However, the studies do not assign proportion of diet to cephalopods or fishes. In the current study, the diet of southern elephant seal is described as favoring cephalopods, with a significant portion of the diet coming from both myctophids and on-shelf fishes.

The seabird functional group represents a diverse group (see S1). There is wide variation in the reported diets of seabirds, though all species feed primarily in the near-surface marine

environment [21-23]. The diet reflects a synthesis of the literature to create a representative diet for this group.

The multispecies groups for cephalopods and on-shelf fish represent diverse species and diets. Cephalopods are known to be opportunistic foragers [24] and important consumers of myctophids and mesopelagic fishes [24, 26], though percentages of diet composition could not be found in the literature. The diet for cephalopods is split between euphausiid and fish groups. All modelled fish groups include krill as significant portions of their diets [27-31, 33, 34]. *Electrona antarctica* serves as the example diet for myctophids as it is the best documented and most abundant myctophid in the region [27, 39].

The diet of Antarctic krill (*Euphausia superba*) varies both by season [40, 42] and life stage [43, 44]. The diets of large and small krill presented here take that variability into account and attempt to reflect an average annual diet for each krill group.

Ballerini et al. [39] re-create monthly diets for non-krill zooplankton based on published and previously non-published data. The diets for other euphausiids, microzooplankton, mesozooplankton, and macrozooplankton are derived from the average diets used by Ballerini et al. (2014). The diet for the other euphausiid group was adjusted to include more microzooplankton based on stable isotope analyses which indicate that other euphausiids occupy a higher trophic level than krill [J. Walsh, Pers. Comm, August 2016]

We assume that the benthic invertebrate group largely consumes detritus that has reached the seafloor. This is consistent with other Antarctic food web models [38, 39, 60]

The initial diet matrix compiled from the literature is provided below. This diet matrix was incrementally adjusted to bring the model into balance.

Table B. Initial unbalanced diet matrix

Model Group	Prey
**Killer Whale**	1% Leopard Seals, 45 % Weddell Seals, 35% Crabeaters Seals, 1% Elephant Seals,1% Blue Whales, 1% Fin Whales, 5% Minke Whales, 1% Humpback Whales, <1% Emperor Penguins, 3% Gentoo Penguins, 3% Chinstrap Penguins, <1% Adélie Penguins, 1% Myctophid fish, 1% On-shelf Fish, <1% *N. rossii*, 1% *G. gibberifrons*
**Leopard Seal**	1% Antarctic Fur Seals, 6% Gentoo Penguins, 5% Chinstrap Penguins, 1% Cephalopods,2% Myctophids, 2% *G. gibberifrons*, 83% Large Krill
**Weddell Seal**	5.5% Cephalopods, 5.5% Myctophids, 86.5% On-shelf Fish, 1.5% *G. gibberifrons*, 1% Benthic Invertebrates
**Crabeater Seal**	1% Cephalopods, 10% Myctophids, 1% On-shelf Fish, 88% Large Krill
**Antarctic Fur Seal**	1% Gentoo Penguins, 1% Chinstrap Penguins, <1% Adélie Penguins, <1% Macaroni Penguins, 2% Cephalopods, 20% Myctophids, 35% On-shelf Fish, 40% Large Krill
**S Elephant Seal**	60% Cephalopods, 20% Myctophids, 10% On-shelf fish, 5% N. rosii, 5% *G. gibberifrons*
**Sperm Whale**	70% Cephalopods, 10% Myctophids, 15% On-shelf Fish, 5% Benthic Invertebrates
**Blue Whale**	70% Large Krill, 10% Other Euphausiids, 20% Macrozooplankton
**Fin Whale**	1% Myctophids, 1 % On-shelf Fish, 70% Large Krill, 10% Other Euphausiids, 5% Mesozooplankton, 13% Macrozooplankton
**Minke Whales**	4% Myctophids, 4% On-shelf fish, 70% Large Krill, 20% Other euphausiids, 2% Macrozooplankton
**Humpback Whale**	1% Cephalopods, 10% Myctophids, 10% On-shelf Fish, 70% Large Krill, 4% Mesozooplankton, 5% Macrozooplankton
**Emperor Penguin**	10% Cephalopods, 38% On-shelf Fish, 52 % Large Krill
**Gentoo Penguin**	15% Myctophids, 15% On-shelf-fish, 70% Large Krill
**Chinstrap Penguin**	5% Myctophids, 4% On-shelf Fish, 90% Large Krill, 1% Macrozooplankton
**Adélie Penguin**	1% Myctophids, <1 % *C. gunnari*, <1% *G. gibberifrons*, 97% Large Krill, 1% Macrozooplankton
**Macaroni Penguin**	1% Cephalopods, 9% Myctophids, 12% On-shelf Fish, 35% Large Krill, 35% Other Euphausiids, 8% Mesozooplankton
**Flying Birds**	30% Cephalopods, 15% Myctophids, 14% On-shelf Fish, 35% Large Krill, 5% Mesozooplankton, 1 % Macrozooplankton
**Cephalopods**	12% Myctophids, 12% On-shelf Fish, 21% Benthic invertebrates, 25% Large Krill, 15% Other Euphausiids, 15% Macrozooplankton
**Myctophids**	40% Large Krill, 20% Other Euphausiids, 20% Mesozooplankton, 20% Macrozooplankton

**On-shelf Fish**	5 % Cephalopods, 10% Myctophids, 1% C. gunnari, 1% Salps, 14% Benthic Invertebrates, 35% Large Krill, 10% Other Euphausiids, 14% Mesozooplankton, 10% Macrozooplankton
N. rossii	10% Myctophids, 5% Salps, 5% Benthic Invertebrates, 50% Large Krill, 20% Other Euphausiids, 10% Ice algae
C. gunnari	5% Myctophids, 80% Large Krill, 5% Other Euphausiids, 10% Macrozooplankton
G. gibberifrons	1% Cephalopods, 1% Myctophids, 1% Salps, 40% Benthic invertebrates, 25 % Large Krill, 2% Macrozooplankton,30% Ice algae
**Salps**	5% Small Krill, 30% Microzooplankton, 5% Mesozooplankton, 30% Small phytoplankton, 30% Large Phytoplankton
**Benthic invertebrates**	100% Detritus
**Large Krill (≥24 months)**	25% Mesozooplankton, 50% Large phytoplankton, 10% Ice Algae, 15% Detritus
**Small Krill (< 24 months)**	15% Microzooplankton, 25% Small phytoplankton, 25% Large phytoplankton, 25% Ice Algae, 10% Detritus
**Other Euphausiids**	30% Mesozooplankton, 50% Large phytoplankton, 20% Detritus
**Microzooplankton**	60% Small phytoplankton, 25% Large phytoplankton, 15% Detritus
**Mesozooplankton**	3% Microzooplankton, 24% Small phytoplankton, 66% Large phytoplankton, 7% Detritus
**Macrozooplankton**	3% Large Krill, 2% Small Krill, 10% Other euphausiids, 70% Mesozooplankton, 5% Small phytoplankton, 10% Large phytoplankton

References

1. Pitman R, Durban J. Killer whale predation on penguins in Antarctica. Polar Biology. 2010;33(11):1589-94. doi: 10.1007/s00300-010-0853-5.

2. Pitman RL, Ensor P. Three forms of killer whales (*Orcinus orca*) in Antarctic waters. Journal of Cetacean Research and Management. 2003;5(2):131-9.

3. Casaux R, Baroni A, Ramón A, Carlini A, Bertolin M, DiPrinzio CY. Diet of the leopard seal *Hydrurga leptonyx* at the Danco Coast, Antarctic Peninsula. Polar Biology. 2009;32(2):307-10. doi: 10.1007/s00300-008-0567-0.

4. Forcada J, Malone D, Royle JA, Staniland IJ. Modelling predation by transient leopard seals for an ecosystem-based management of Southern Ocean fisheries. Ecological Modelling. 2009;220(12):1513-21. doi: http://dx.doi.org/10.1016/j.ecolmodel.2009.03.020.

5. Boveng PL, Hiruki LM, Schwartz MK, Bengtson JL. Population growth of Antarctic fur seals: limitation by a top predator, the leopard seal? Ecology. 1998;79(8):2863-77. doi: 10.2307/176522.

6. Casaux R, Baroni A, Ramón A. The diet of the Weddell seal *Leptonychotes weddellii* at the Danco Coast, Antarctic Peninsula. Polar Biology. 2006;29(4):257-62. doi: 10.1007/s00300-005-0048-7.

7. Hückstädt L, Burns J, Koch P, McDonald B, Crocker D, Costa D. Diet of a specialist in a changing environment: the crabeater seal along the western Antarctic Peninsula. Marine Ecology Progress Series. 2012;455:287-301. doi: 10.3354/meps09601.

8. Casaux R, Baroni A, Ramón A. Diet of Antarctic fur seals *Arctocephalus gazella* at the Danco Coast, Antarctic Peninsula. Polar Biology. 2003;26(1):49-54. doi: 10.1007/s00300-002-0442-3.

9. Polito MJ, Goebel ME. Investigating the use of stable isotope analysis of milk to infer seasonal trends in the diets and foraging habitats of female Antarctic fur seals. Journal of Experimental Marine Biology and Ecology. 2010;395(1–2):1-9. doi: http://dx.doi.org/10.1016/j.jembe.2010.08.015.

10. Hückstädt LA, Koch PL, McDonald BI, Goebel ME, Crocker DE, Costa DP. Stable isotope analyses reveal individual variability in the trophic ecology of a top marine predator, the southern elephant seal. Oecologia. 2012;169(2):395-406. doi: 10.1007/s00442-011-2202-y.

11. Pauly D, Trites AW, Capuli E, Christensen V. Diet composition and trophic levels of marine mammals. ICES Journal of Marine Science: Journal du Conseil. 1998;55(3):467-81. doi: 10.1006/jmsc.1997.0280.

12. Kawamura A. An interim consideration on a possible insterpecific relation in southern baleen whales from the viewpoint of their food habits. Report of the International Whaling Commission. 1978;28:411-20.

13. Armstrong AJ, Siegfried WR. Consumption of Antarctic krill by minke whales. Antarctic Science. 1991;3(01):13-8. doi: doi:10.1017/S0954102091000044.

14. Santora J, Schroeder I, Loeb V. Spatial assessment of fin whale hotspots and their association with krill within an important Antarctic feeding and fishing ground. Mar Biol. 2014;161(10):2293-305. doi: 10.1007/s00227-014-2506-7.

15. Klages N. Food and feeding ecology of emperor penguins in the eastern Weddell Sea. Polar Biology. 1989;9(6):385-90. doi: 10.1007/BF00442529.

16. Cherel Y, Kooyman GL. Food of emperor penguins (*Aptenodytes forsteri*) in the western Ross Sea, Antarctica. Mar Biol. 1998;130(3):335-44. doi: 10.1007/s002270050253.

17. Miller AK, Kappes MA, Trivelpiece SG, Trivelpiece WZ. Foraging-niche separation of breeding gentoo and chinstrap penguins, South Shetland Islands, Antarctica. Condor. 2010;112(4):683-95. doi: 10.1525/cond.2010.090221. PubMed PMID: WOS:000285727700007.

18. Polito MJ, Trivelpiece WZ, Karnovsky NJ, Ng E, Patterson WP, Emslie SD. Integrating stomach content and stable isotope analyses to quantify the diets of pygoscelid penguins. PLoS One. 2011;6(10):e26642. doi: 10.1371/journal.pone.0026642. PubMed PMID: PMC3203888.

19. Lynnes AS, Reid K, Croxall JP. Diet and reproductive success of Adélie and chinstrap penguins: linking response of predators to prey population dynamics. Polar Biology. 2004;27(9):544-54. doi: 10.1007/s00300-004-0617-1.

20. Deagle BE, Gales NJ, Evans K, Jarman SN, Robinson S, Trebilco R, et al. Studying seabird diet through genetic analysis of faeces: a case study on macaroni penguins (*Eudyptes chrysolophus*). PLoS One. 2007;2(9):e831. doi: 10.1371/journal.pone.0000831. PubMed PMID: PMC1959119.

21. Ainley DG, Ribic CA, Fraser WR. Ecological structure among migrant and resident seabirds of the Scotia--Weddell confluence region. Journal of Animal Ecology. 1994;63(2):347-64. doi: 10.2307/5553.

22. Ainley DG, O'Connor EF, Boekelheide RJ. The marine ecology of birds in the Ross Sea, Antarctica. Ornithological Monographs. 1984;(32):iii-97. doi: 10.2307/40166773.

23. Malzof S, Quintana R. Diet of the south polar skua *Catharacta maccormicki* and the brown skua *C. antarctica lonnbergi* at Cierva Point, Antarctic Peninsula. Polar Biology. 2008;31(7):827-35. doi: 10.1007/s00300-008-0421-4.

24. Rodhouse PG, Nigmatullin CM. The role of cephalopods in the world's oceans- role as consumers. Philosophical Transactions: Biological Sciences. 1996;351(1343):1003-22. doi: 10.2307/56292.

25. Phillips KL, Jackson GD, Nichols PD. Predation on myctophids by the squid *Moroteuthis ingens* around Macquarie and Heard Islands: stomach contents and fatty acid analyses. Marine Ecology Progress Series. 2001;215:179-89. doi: 10.3354/meps215179.

26. Kozlov AN. A review of the trophic role of mesopelagic fish of the family *myctophidae* in the Southern Ocean ecosystem. CCAMLR Science. 1995;2:71-7.

27. Pusch C, Hulley PA, Kock KH. Community structure and feeding ecology of mesopelagic fishes in the slope waters of King George Island (South Shetland Islands, Antarctica). Deep Sea Research Part I: Oceanographic Research Papers. 2004;51(11):1685-708. doi: http://dx.doi.org/10.1016/j.dsr.2004.06.008.

28. Pakhomov E, Perissinotto R, McQuaid C. Prey composition and daily rations of myctophid fishes in the Southern Ocean. Marine Ecology Progress Series. 1996;134:1-14. doi: 10.3354/meps134001.

29. La Mesa M, Eastman JT, Vacchi M. The role of notothenioid fish in the food web of the Ross Sea shelf waters: a review. Polar Biology. 2004;27(6):321-38. doi: 10.1007/s00300-004-0599-z.

30. Barrera-Oro E. The role of fish in the Antarctic marine food web: differences between inshore and offshore waters in the southern Scotia Arc and west Antarctic Peninsula. Antarctic Science. 2002;14(04):293-309.

31. Casaux R, Barrera-Oro E. Dietary overlap in inshore notothenioid fish from the Danco Coast, western Antarctic Peninsula. Polar Research. 2013;32:1-8. PubMed PMID: 1503556968; ZOOR15003010155.

32. Jones CD, Ashford J, Dietrich K, DeVries A, Hanchet S, Kock KH, et al. Bottom Trawl Survey. In: Lipsky JD, editor. AMLR 2002/2003 Field season report: objectives, accomplishments and tentative conclusions. NOAA-TM-NMFS-SWFSC-355. La Jolla, California: U.S. Department of Commerce, National Oceanic and Atmospheric Administration, National Marine Fisheries Service, Southwest Fisheries Science Center, Antarctic Ecosystem Research Division; 2003. p. 111-43. Available at: https://swfsc.noaa.gov/textblock.aspx?Division=AERD&id=3154&ParentMenuId=42.

33. Main C, Collins M, Mitchell R, Belchier M. Identifying patterns in the diet of mackerel icefish (*Champsocephalus gunnari*) at South Georgia using bootstrapped confidence intervals of a dietary index. Polar Biology. 2009;32(4):569-81. doi: 10.1007/s00300-008-0552-7.

34. Flores H, Kock KH, Wilhelms S, Jones CD. Diet of two icefish species from the South Shetland Islands and Elephant Island, *Champsocephalus gunnari* and *Chaenocephalus aceratus*. Polar Biology. 2004;27(2):119-29. doi: 10.1007/s00300-003-0570-4.

35. Jones CD, Brooks CM, Dietrich B, Dietrich K, Driscoll R, Kendrick J, et al. Demersal finfish survey of the Northern Antarctic Peninsula In: Lipsky JD, editor. AMLR 2005/2006 Field season report: objectives, accomplishments, and tentative conclusions. NOAA-TM-NMFS-SWFSC-397. La Jolla, California: U.S. Departmetn of Commerce, National Oceanic and Atmospheric Administration, National Marine Fisheries Service, Southwest Fisheries Science Center, Antarctic Ecosystem Research Division; 2006. p. 107-34. Avaialable at: https://swfsc.noaa.gov/textblock.aspx?Division=AERD&id=3154&ParentMenuId=42.

36. Pakhomov EA, Dubischar CD, Strass V, Brichta M, Bathmann UV. The tunicate *Salpa thompsoni* ecology in the Southern Ocean. I. Distribution, biomass, demography and feeding ecophysiology. Mar Biol. 2006;149(3):609-23. doi: 10.1007/s00227-005-0225-9.

37. Perissinotto R, A. Pakhomov E. The trophic role of the tunicate *Salpa thompsoni* in the Antarctic marine ecosystem. Journal of Marine Systems. 1998;17(1–4):361-74. doi: http://dx.doi.org/10.1016/S0924-7963(98)00049-9.

38. Jarre-Teichmann A, Brey T, Bathmann U, Dahm C, Dieckmann G, Gorny M, et al. Trophic flows in the benthic shelf community of the eastern Weddell Sea, Antarctica. In: Battaglia B,

Valencia J, Walton DWH, editors. Antarctic communities: species, structure and survival. Cambridge: Cambridge University, Press; 1997. p. 118-34.

39. Ballerini T, Hofmann EE, Ainley DG, Daly K, Marrari M, Ribic CA, et al. Productivity and linkages of the food web of the southern region of the western Antarctic Peninsula continental shelf. Prog Oceanogr. 2014;122(0):10-29. doi: http://dx.doi.org/10.1016/j.pocean.2013.11.007.

40. Atkinson A, Meyer B, Stuϋbing D, Hagen W, Schmidt K, Bathmann UV. Feeding and energy budgets of Antarctic krill *Euphausia superba* at the onset of winter—II. Juveniles and adults. Limology and Oceanography. 2002;47(4):953-66. doi: https://doi.org/10.4319/lo.2002.47.4.0953.

41. Atkinson A, Snÿder R. Krill-copepod interactions at South Georgia, Antarctica, I. Omnivory by *Euphausia superba*. Marine Ecology Progress Series. 1997;160:63-76. doi: 10.3354/meps160063.

42. Perissinotto R, Pakhomov EA, McQuaid CD, Froneman PW. *In situ* grazing rates and daily ration of Antarctic krill *Euphausia superba* feeding on phytoplankton at the Antarctic Polar Front and the marginal ice zone. Marine Ecology Progress Series. 1997;160:77-91. doi: 10.3354/meps160077.

43. Meyer B, Atkinson A, Blume B, Bathmann UV. Feeding and energy budgets of larval Antarctic krill *Euphausia superba* in summer. Marine Ecology Progress Series. 2003;257:167-78. doi: 10.3354/meps257167.

44. Meyer B, Atkinson A, Stübing D, Oettl B, Hagen W, Bathmann U. Feeding and energy budgets of Antarctic krill *Euphausia superba* at the onset of winter—I. furcilia III larvae. Limology and Oceanography. 2002;47(4):943-52. doi: https://doi.org/10.4319/lo.2002.47.4.0943.

45. Töbe K, Meyer B, Fuentes V. Detection of zooplankton items in the stomach and gut content of larval krill, *Euphausia superba*, using a molecular approach. Polar Biology. 2010;33(3):407-14. doi: 10.1007/s00300-009-0714-2.

46. Ross RM, Quetin LB, Baker KS, Vernet M, Smith RC. Growth limitation in young *Euphausia superba* under field conditions. Limology and Oceanography. 2000;31-43. doi: https://doi.org/10.4319/lo.2000.45.1.003.

47. Hopkins TL. Food web of an Antarctic midwater ecosystem. Mar Biol. 1985;89(2):197-212. doi: 10.1007/BF00392890.

48. Marrari M, Daly KL, Timonin A, Semenova T. The zooplankton of Marguerite Bay, Western Antarctic Peninsula—Part I: Abundance, distribution, and population response to variability in environmental conditions. Deep Sea Research Part II: Topical Studies in Oceanography. 2011;58(13–16):1599-613. doi: http://dx.doi.org/10.1016/j.dsr2.2010.12.007.

49. Donnelly J, Sutton T, Torres J. Distribution and abundance of micronekton and macrozooplankton in the NW Weddell Sea: relation to a spring ice-edge bloom. Polar Biology. 2006;29(4):280-93. doi: 10.1007/s00300-005-0051-z.

50. Froneman P, Perissinotto R. Microzooplankton grazing in the Southern Ocean: implications for the carbon cycle. Marine Ecology. 1996;17(1-3):99-115. doi: 10.1111/j.1439-0485.1996.tb00493.x.

51. Froneman PW, Pakhomov EA, Perissinotto R, McQuaid CD. Role of microplankton in the diet and daily ration of Antarctic zooplankton species during austral summer. Marine Ecology Progress Series. 1996;143:15-23. doi: 10.3354/meps143015.

52. Pasternak A, Schnack-Schiel S. Seasonal feeding patterns of the dominant Antarctic copepods *Calanus propinquus* and *Calanoides acutus* in the Weddell Sea. Polar Biology. 2001;24(10):771-84. doi: 10.1007/s003000100283.

53. Swadling KM, Gibson JAE, Ritz DA, Nichols PD, Hughes DE. Grazing of phytoplankton by copepods in eastern Antarctic coastal waters. Mar Biol. 1997;128(1):39-48. doi: 10.1007/s002270050066.

54. Pakhomov EA, Froneman PW. Zooplankton dynamics in the eastern Atlantic sector of the Southern Ocean during the austral summer 1997/1998—Part 2: grazing impact. Deep Sea Research Part II: Topical Studies in Oceanography. 2004;51(22–24):2617-31. doi: http://dx.doi.org/10.1016/j.dsr2.2000.11.002.

55. Øresland V, Ward P. Summer and winter diet of four carnivorous copepod species around South Georgia. Marine Ecology Progress Series. 1993;98(1-2):73-8. doi: https://doi.org/10.3354/meps098073.

56. Pakhomov E, Perissinotto R. Trophodynamics of the hyperiid amphipod *Themisto gaudichaudi* in the South Georgia region during late austral summer. Marine Ecology Progress Series. 1996;134:91-100. doi: 10.3354/meps134091.

57. Goebel ME, Reiss C. Squeezed from both ends: decline in Antarctic fur seals in the South Shetland Islands driven by both top–down and bottom–up processes. 2014:WG-EMM-14-39; data available at https://data.nodc.noaa.gov/cgi-bin/iso?id=gov.noaa.nodc:0186008.

58. Daneri G, Carlini A. Fish prey of southern elephant seals, *Mirounga leonina*, at King George Island. Polar Biology. 2002;25(10):739-43. doi: 10.1007/s00300-002-0408-5.

59. Daneri GA, Carlini AR, Rodhouse PGK. Cephalopod diet of the southern elephant seal, *Mirounga leonina*, at King George Island, South Shetland Islands. Antarctic Science. 2000;12(01):16-9.

60. Hoover C, Pitcher T, Pakhomov E. The Antarctic Peninsula Marine Ecosystem Model and Simulations: 1978- Present. In: Wabnitz CC, Hoover C, editors. From the tropics to the poles:

ecosystem models of Hudson Bay, Kaloko-Honokohau, Hawai'i, and the Antarctic Peninsula, Fisheries Centre Research Reports 20(2). Unversity of British Columbia: Fisheries Centre; 2012. p. 108-82.
