# Supplementary material for: Using sea-ice to calibrate a dynamic trophic model for the Western Antarctic Peninsula
Source: PLoS One. 2019 Apr 2;14(4):e0214814. doi: 10.1371/journal.pone.0214814 (PMC6445414; doi:10.1371/journal.pone.0214814)
Supplement: S6 File — (PDF) [file pone.0214814.s006.pdf]

S6 File. Time series sources

Table Data sources for the time-series describing biomass trends

Model Groups	Time Series Source(s)	Notes
**Antarctic fur seal**	Goebel and Reiss [1]	Annual pup count at the largest fur seal colony in the region. Pup production at Cape Shireff represents approximately 80% of pups produced in region.
**Adélie Penguin**	Hinke et al. [2], [3] and LTER (http://oceaninformatics.ucsd.edu/datazoo/data/pallter/datasets)	Count from the colonies monitored in the LTER study area were added to those monitored by NOAA's U.S.AMLR Program
**Chinstrap Penguin**	Hinke et al. [2], [3]	Colony counts from NOAA's U.S. AMLR Program
**Gentoo Penguin**	Hinke et al. [2], [3]	Colony counts from NOAA's U.S. AMLR Program
N. rossii	Kock and Jones (2005) and Kock and Jones [4]	The time series was created by combining early published data with more recent data submitted to CCAMLR.
C. gunnari	Kock and Jones (2005) and Kock and Jones [4]	The time series was created by combining early published data with more recent data submitted to CCAMLR.
G. gibberifrons	Kock and Jones (2005) and Kock and Jones [4]	The time series was created by combining early published data with more recent data submitted to CCAMLR.
**Large Krill**	NOAA-AMLR (https://swfsc.noaa.gov/AERD-Data/)	This time series represents average annual krill desinties estimated from systematic acoustic surveys.

References

1. Goebel ME, Reiss C. Squeezed from both ends: decline in Antarctic fur seals in the South Shetland Islands driven by both top–down and bottom–up processes. 2014:WG-EMM-14-39; data available at https://data.nodc.noaa.gov/cgi-bin/iso?id=gov.noaa.nodc:0186008.

2. Hinke JT, Salwicka K, Trivelpiece SG, Watters GM, Trivelpiece WZ. Divergent responses of Pygoscelis penguins reveal a common environmental driver. Oecologia. 2007;153(4):845-55. doi: 10.1007/s00442-007-0781-4. PubMed PMID: WOS:000249407000006.

3. Hinke J. US NOAA AMLR Program penguin colony counts. https://data.nodc.noaa.gov/cgi-bin/iso?id=gov.noaa.nodc:01851132018.

4. Kock KH, Jones CD. The composition, abundance and reproductive characteristics of the demersal fish fauna in the Elephant Island–South Shetland Islands region and at the tip of the Antarctic Peninsula (CCAMLR Subarea 48.1) in March–early April 2012. 2012:WG-FSA-12-0. Paper available by e-mail to chris.d.jones@noaa.gov.
