# Supplementary material for: Using sea-ice to calibrate a dynamic trophic model for the Western Antarctic Peninsula
Source: PLoS One. 2019 Apr 2;14(4):e0214814. doi: 10.1371/journal.pone.0214814 (PMC6445414; doi:10.1371/journal.pone.0214814)

S7 File. Calibrated model results for groups without time series data

Figure A. Cetaceans

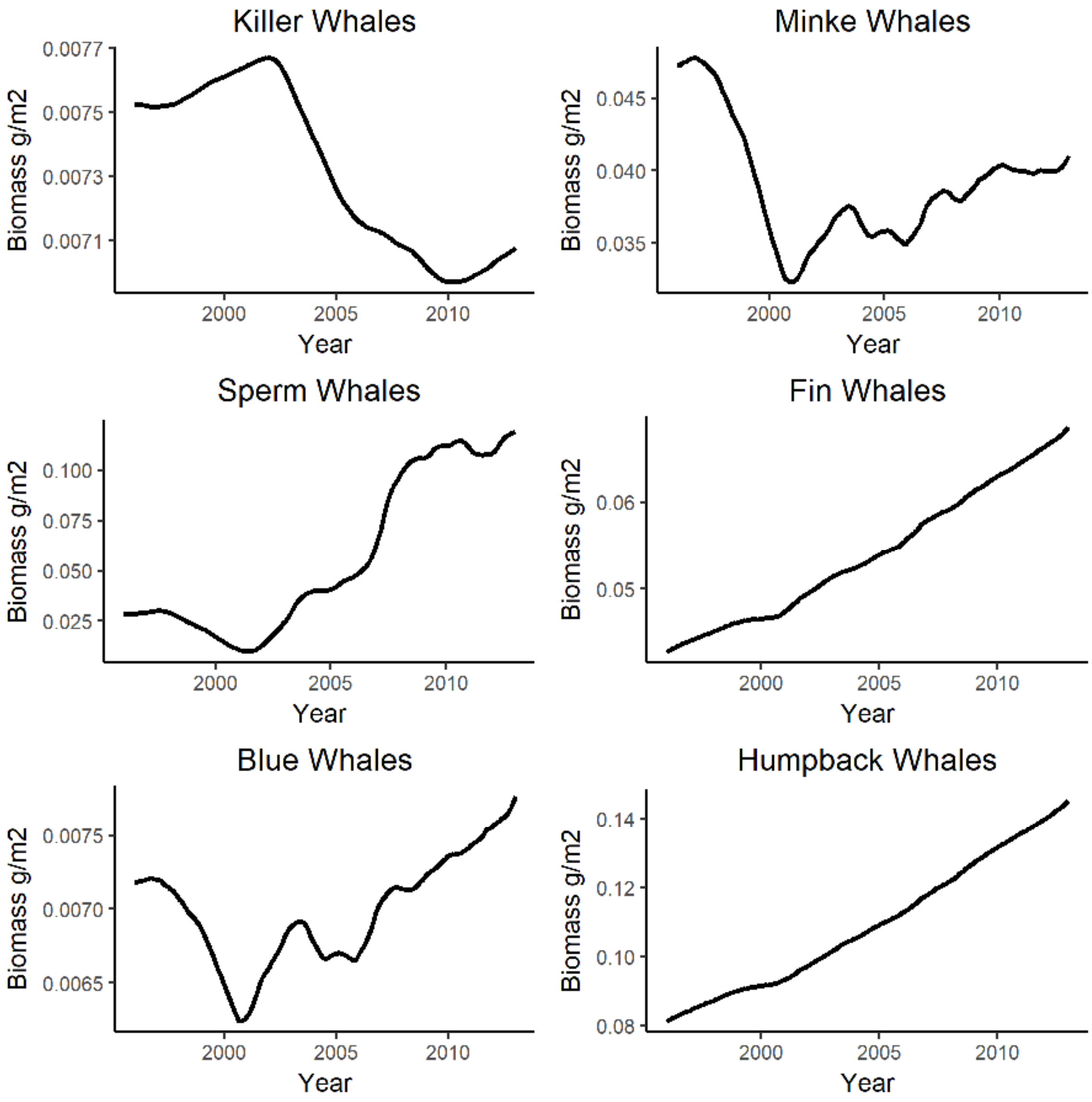

Figure B. Seals and birds

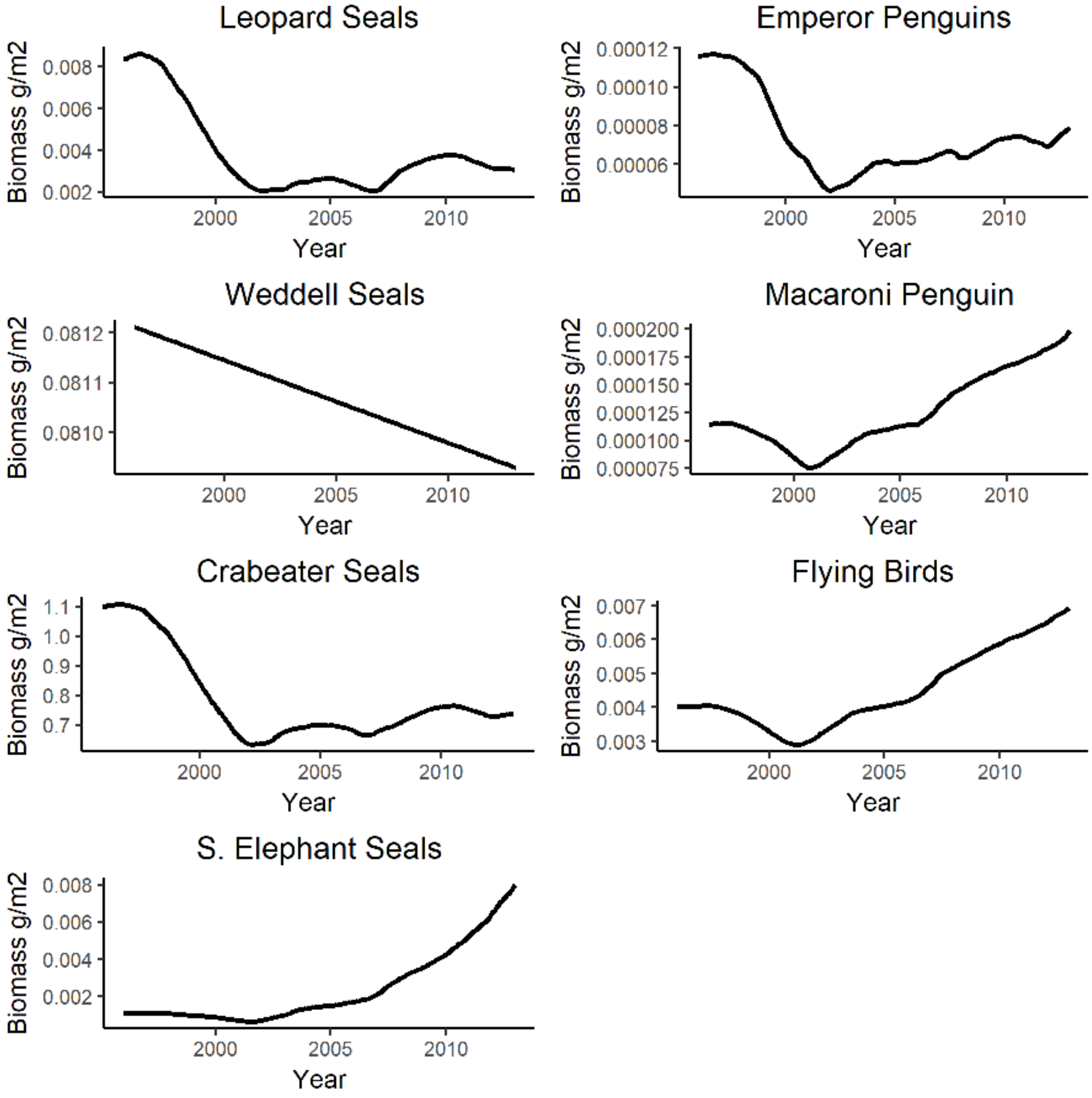

Figure C. Fish, cephalopods, and euphausiids

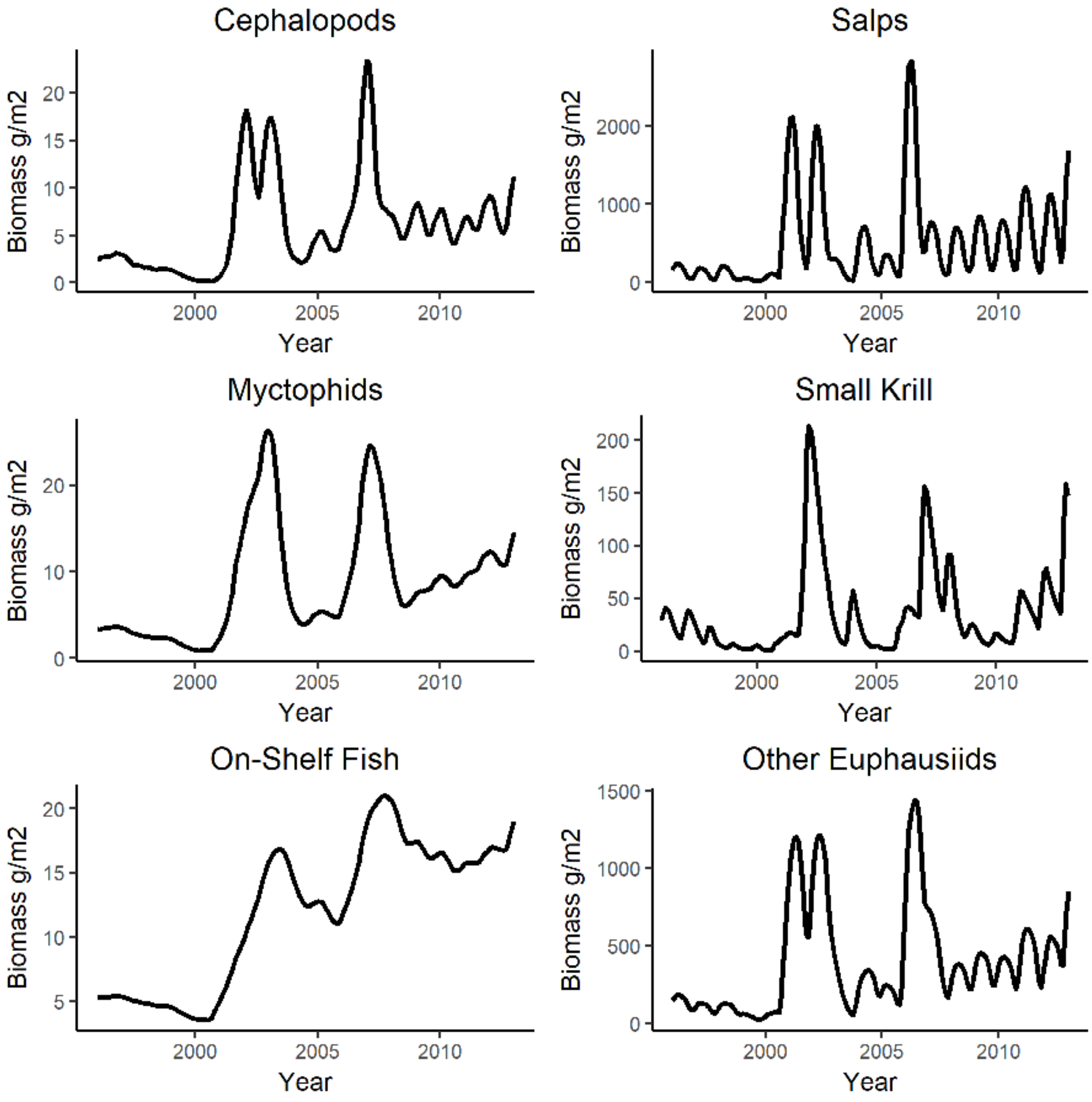

Figure D. Benthic invertebrates, size-classed plankton and detritus

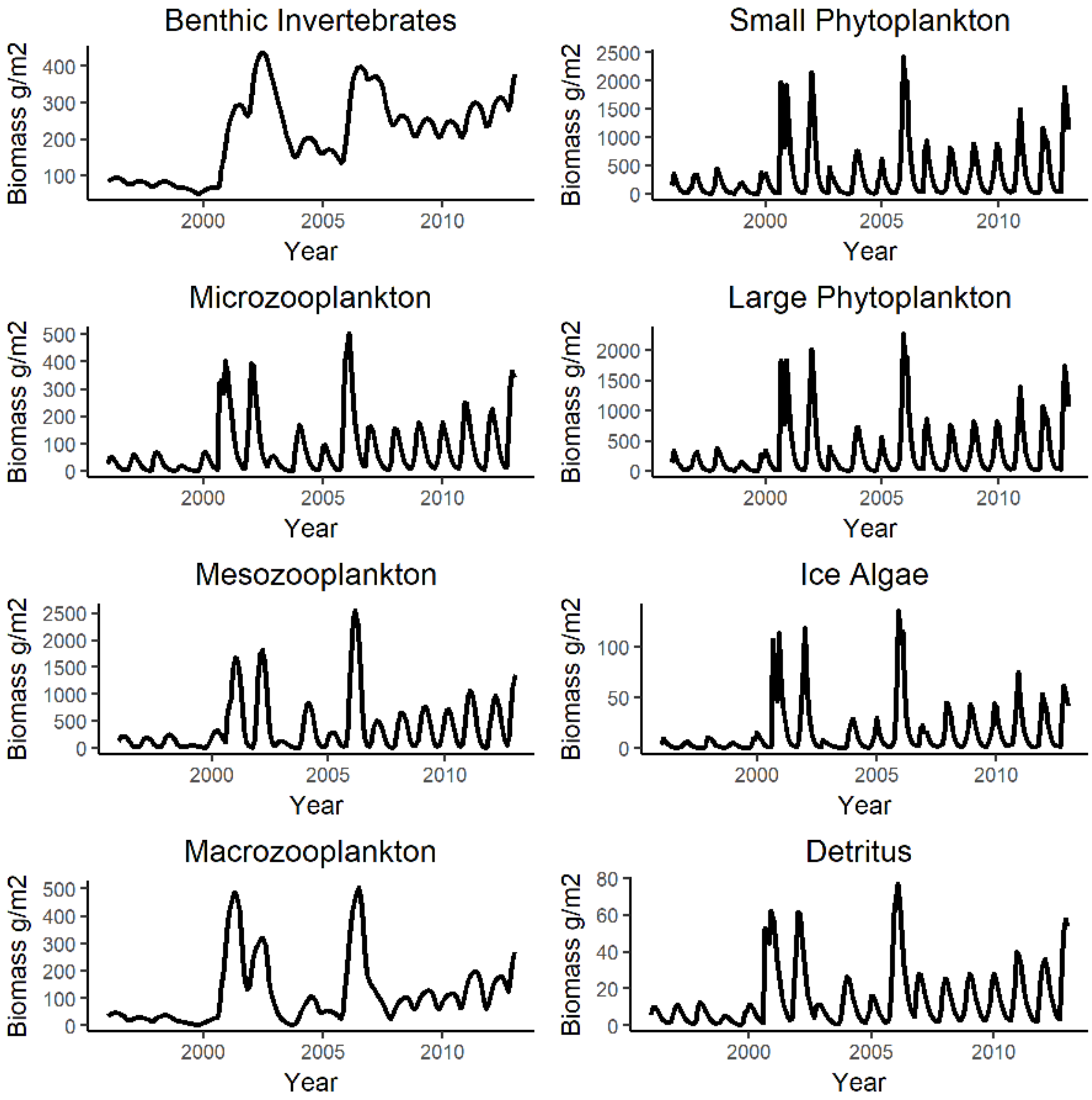

Supplement: S7 File — (PDF) [file pone.0214814.s007.pdf]
