# Supplementary figures and images for: Using sea-ice to calibrate a dynamic trophic model for the Western Antarctic Peninsula
Source: PLoS One. 2019 Apr 2;14(4):e0214814. doi: 10.1371/journal.pone.0214814 (PMC6445414; doi:10.1371/journal.pone.0214814)

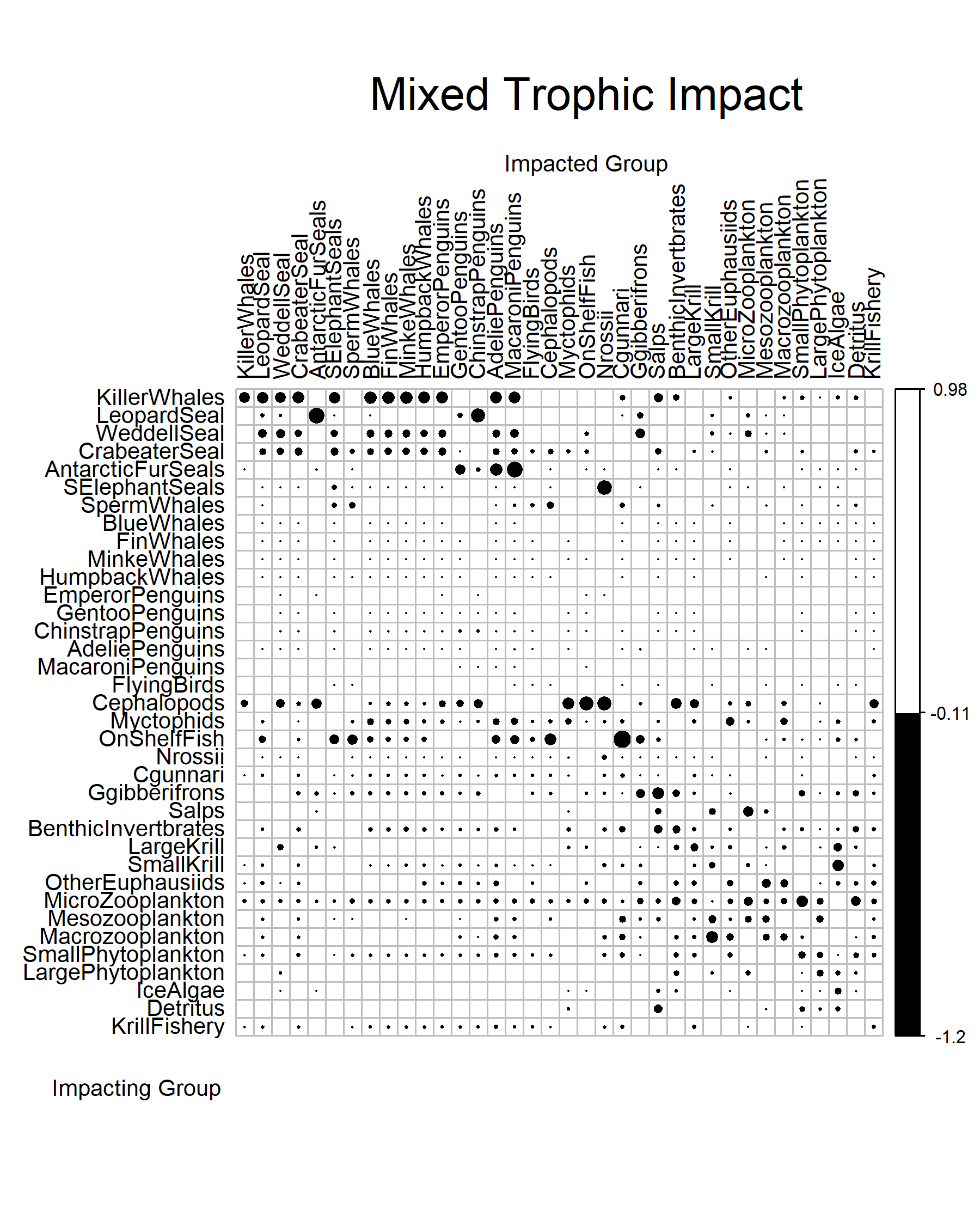

Supplement: S8 File — Black indicates a negative impact, white indicates a positive impact; size of the circle indicates strength of the impact. (TIF) [file pone.0214814.s008.tif]
